# Sweet Potato New Varieties Screening Based on Morphology, Pulp Color, Proximal Composition, and Total Dietary Fiber Content *via* Factor Analysis and Principal Component Analysis

**DOI:** 10.3389/fpls.2022.852709

**Published:** 2022-05-05

**Authors:** Cláudio Eduardo Cartabiano Leite, Brunna de Kácia Ferreira Souza, Candida Elisa Manfio, Gerson Henrique Wamser, Daniel Pedrosa Alves, Alicia de Francisco

**Affiliations:** ^1^Cereal Science and Technology Laboratory, Food Science Post-Graduation Program (PPGCAL), Agrarian Sciences Center, Federal University of Santa Catarina (UFSC), Florianópolis, Brazil; ^2^Cereal Science and Technology Laboratory, Food Science and Technology Department (CTA), Agrarian Sciences Center, Federal University of Santa Catarina (UFSC), Florianópolis, Brazil; ^3^Agricultural Research and Rural Extension of Santa Catarina (EPAGRI) – Ituporanga Experimental Station, Ituporanga, Brazil

**Keywords:** sweet potato, new crop variety, total dietary fiber, factor analysis, principal component analysis

## Abstract

A sample set of 18 sweet potatoes [*Ipomoea batatas* (L.) Lam] segmented into six registered cultivars and 12 new varieties were evaluated. The 142 tuberous roots were obtained from a sweet potato germplasm bank (BAG-sweet potato; -27.417713768824555 and -49.64874168439556), specifically from plants belonging to a sweet potato breeding program. All samples were characterized according to their morphology, instrumental pulp color, proximate composition, and total dietary fiber. The analytical results were submitted to parametric and non-parametric statistical tests for sample variance data comparison. Moreover, the screening of the cultivars and new varieties was performed by exploratory statistical analysis, factor analysis (FA), and principal component analysis (PCA). From the sixteen independent variables that characterized the samples, the exploratory FA identified thirteen that had a communality greater than 0.7, with 92.08% of assertiveness. The PCA generated 4 principal components able to account for 84.01% of the explanatory variance. So, among the six registered cultivars, SCS372 Marina and SCS370 Luiza showed the capability to be employed as cultivars for production. Among the 12 sweet potato new varieties, samples 17025-13, 17125-10, and 17117 met the requirements for patent and registration. These results will be useful to farmers who wish to use these sweet potatoes in the development of their crops.

## Introduction

Sweet potato [*Ipomoea batatas* (L.) Lam] is a tuberous root known worldwide as a valuable food crop. The plant belongs to the Convolvulaceae family, and it is the only specimen among its peers of the genus Ipomoea capable of producing tuberous roots that are consumed as a staple food and employed as industrial raw material in many processes (Meira et al., 2012; Mu et al., 2017; Singh, 2019).

Sweet potato is an important food crop for the food security of the inhabitants of tropical and subtropical countries. The consumption of its tuberous roots, as well as the plant leaves, can assist in mitigating negative health impacts caused by the high incidence of malnutrition, particularly among women, pregnant women, and children (Wadl et al., 2018; Amagloh et al., 2021; Gasura et al., 2021). Various studies report the beneficial effects that sweet potato components can have on human metabolisms, such as anti-inflammatory (Chen et al., 2019), anticancer (Li et al., 2013; Mohanraj and Sivasankar, 2014; Sugata et al., 2015), antidiabetic (Dutta, 2015; Yuan et al., 2017), and anti-obesity (Ju et al., 2011; Kim et al., 2020) effects. Notably, the consumption of orange-fleshed sweet potatoes is related to the vitamin A increase in the body (Van Jaarsveld et al., 2005; Low et al., 2007). So, researchers recommend the daily consumption of sweet potatoes to stimulate beneficial health effects (Wang et al., 2016; Alam, 2021; Amagloh et al., 2021).

Most varieties of sweet potatoes have a moisture content of 60–80%. Hence, total solids correspond to 20–40% of the total composition (Rose and Vasanthakaalam, 2011; Júnior et al., 2012; Ruttarattanamongkol et al., 2016; Sun et al., 2017; Heo et al., 2021). The average composition of the pulp includes 43.5% total starch, 2% protein, 0.4% lipids, 4.4% ash, and 49.7% total dietary fiber (Mu and Singh, 2019). Furthermore, the sweet potato cultivars known as color pulp varieties possess bioactive compounds in their composition, such as phenolic acids, anthocyanins, and carotenoids (Alam, 2021).

Agricultural data made available by the Food and Agriculture Organization (FAO) indicate that in 2019, sweet potatoes were produced in 109 countries, with China (mainland) standing out as the largest producer with a harvest of more than 51 million tons of tuberous roots. Among the main sweet potato producers in 2019 are the countries that make up the African and Asian continents, as well as the United States of America (Food and Agriculture Organization of the United Nations, 2021).

Some crop features such as economic prominence, high yield, and nutritional quality of the tuberous roots suggest further research for plant genetic improvement (Gasura et al., 2021). Furthermore, sweet potato genetic improvement programs aim to provide new plant varieties for the farmers, consumers, and industry with unique characteristics to satisfy the characteristic demands of each sector (Grüneberg et al., 2015; Mu et al., 2017; Cartabiano et al., 2020; Low et al., 2020).

In their extensive review, Katayama et al. (2017) highlighted several issues that sweet potato genetic breeding programs face, such as achieving new plant varieties that provide a higher production yield, good morphological characteristics, resistance to drought and climate change, adaptation to the management of direct planting, and resistance to pests that affect the crop and stored tubers. Regarding structural and nutritional composition, the tuberous roots should present morphological quality, higher nutrient content, total dietary fiber, starch, and bioactive compounds (Tanaka et al., 2017).

If the agricultural producer does not invest in farming new varieties of sweet potatoes, both consumers and the food industry will not find the tuberous roots produced by better quality plants (Federizzi et al., 2012). Therefore, studies that aim to assess different demands required by agents that work with sweet potatoes are essential. This is crucial to support a joint action plan to improve future crops (Labrada, 2009; De Jonge et al., 2021).

In general, farmers have difficulties in accessing scientific publications and information that can corroborate the quality of the new food plant varieties that arise from genetic improvement programs. Scientific communications that highlight research based on the different demands from the productive agricultural sector can mitigate that gap (Weltzien et al., 2019; Qaim, 2020). Published research reports should aim at providing support for the decision-making of growers related to the advantages of establishing sweet potato crops based on the use of new plant varieties (Katayama et al., 2017; Singh et al., 2021).

Likewise, the consumers of sweet potatoes have particular requirements regarding the nutritional quality of the roots, associated with shape and appearance. Furthermore, demanding nutritional consumers are also aware that sweet potatoes with colored pulp, especially orange-fleshed and purple-fleshed sweet potato, own bioactive compounds in their composition that are beneficial to their health (Truong et al., 2018; Ricachenevsky et al., 2019; Low et al., 2020; Mwanga et al., 2021).

Therefore, the purpose of this study was to provide information to support the farmer’s decisions to invest in the agricultural production of new sweet potato varieties. The main objective was to evaluate the morphological quality, pulp color differences, proximate composition, and the total dietary fiber content in patented cultivars and new varieties of sweet potatoes that were obtained by a genetic plant breeding program. The analytical results will be used for screening among the sweet potato crops.

## Materials and Methods

### Sample Collection

Registered cultivars and new varieties of sweet potatoes from the 2020 harvest were developed by the Agricultural Research and Rural Extension Company – Ituporanga Experimental Station, Santa Catarina, Brazil. The tuberous roots were collected from the Active Sweet Potato Germplasm Bank (BAG-batata-doce; −27.417713768824555, −49.64874168439556). From 140 genotypes cultivated in the germplasm bank, 18 samples were subdivided into 6 registered cultivars, and 12 prominent new varieties were chosen. The sweet potatoes acquired were randomly harvested to avoid bias. Three plants of each cultivar were selected from the growing field, and the tuberous roots produced by each plant were used for the study. Table 1 shows the sample number, names, and quantity of tuberous roots evaluated per sample. It can be observed that all plants did not produce the same number of roots which could be related to genetic and environmental factors.

**TABLE 1:** New sweet potato varieties and registered samples.

Number	Name	Roots analyzed by sample	Codification
1	SCS367 Favorita	18	27465*
2	SCS368 Ituporanga	11	27464*
3	SCS369 Águas Negras	9	27463*
4	SCS370 Luiza	13	32952*
5	SCS371 Katiy	10	32953*
6	SCS372 Marina	9	32954*
7	Darci	6	New variety
8	Leandro	3	New variety
9	17007-15	8	New variety
10	17025-13	9	New variety
11	17082-8	6	New variety
12	17092-9	5	New variety
13	17105-20	7	New variety
14	17107-18	5	New variety
15	17125-10	5	New variety
16	17052	6	New variety
17	17117	7	New variety
18	17162	5	New variety

*Sweet potatoes registered in National Cultivars Registry, of The Department of Agriculture, Livestock and Food Supply, Brazil.

### Sample Preparing

Sweet potato samples were collected and conditioned in unitary kraft paper before being transported to the Department of Food Science and Technology at the Federal University of Santa Catarina (UFSC, Brazil). The tuberous roots were cleaned and sanitized with sodium hypochlorite solution (200 ppm) and rinsed in distilled water followed by overnight drying at room temperature. Some tuberous root units from each sample were freshly analyzed, and others were vacuum-packed with embossed Nylon-Poli packaging (Registron, São Paulo, Brazil) followed by storage in a freezer at a temperature of −20°C.

#### Pulp Freeze-Drying

For specific analyses with tuberous roots on a dry basis, the samples at −20°C were fast transferred to an ultra-freezer (Nuaire Glacier Blue) (NuAire, Plymouth, United States) and stored at −55°C. Those samples were peeled, and the pulp was lyophilized with vacuum for 48 h (Terroni LS3000D) (Terroni Scientific Equipment, São Carlos, Brazil) at Multiuser Laboratory for Biological Studies (LAMEB, Brazil). After freeze-drying, the dried pulp was finely ground in a hammer mill, packed in falcon tubes, and stored in a desiccator at room temperature.

### Morphological Analysis

Morphology was evaluated according to the protocol established by the Company of Warehouses and General Storehouses of São Paulo (CEAGESP, Brazil). Tuberous roots were examined according to morphological traits, the visual color of primary and secondary skin, weight (g), length (cm), and width (cm). Weight was obtained using a Marte A1600 semi-analytical scale (Marte Científica, São Paulo, Brazil) (±<0.01 g), and length and width were evaluated with a Digimess Stainless-Hardened caliper (Digimess Precision Instruments, São Paulo, Brazil) (Coordenadoria De Desenvolvimento Dos Agronegócios [CODEAGRO], 2014). The morphological descriptors of tuberous roots are in agreement with Huaman (1991).

### Instrumental Color

The instrumental color assessment was performed in fresh samples using a portable Minolta CR-400 colorimeter (light source D65) (Konica Minolta Sensing Americas, New Jersey, United States). The following parameters were obtained: L* (Luminosity; 0 black and 100 white), a* (+a* = red; −a* = green) and b* (+b* = yellow; −b* = blue). The factors C* (saturation) and h° (hue angle) were obtained with equations 1 and 2, as recommended by Konica Minolta Corporation (1994). All instrumental color results were evaluated and interpreted using the CIELAB (Caivano and Buera, 2016) and CIEL*C*h (Konica Minolta Corporation, 2017) systems.


(1) $$C^{*}=\sqrt{{\left(a^{*}\right)}^{2}+{\left(b^{*}\right)}^{2}}$$

(2) $$h^{{}^{\circ}}=t\,a\,n^{-1}\,\left(\frac{b^{*}}{a^{*}}\right)$$

### Proximal Composition

#### Moisture

Initially, samples of fresh pulp were fragmented and submitted to the split technique to form the sample set. Analysis was performed according to the Association of Agricultural Chemists (AOAC) method 934.01 (Association of Agricultural Chemists [AOAC], 2005). The samples were crushed into 5 g and placed in a weighted crucible previously dried in an oven. The crucibles with the samples were subjected to drying at 105°C to constant weight. The samples were then removed, weighed, and moisture percentage was determined.

#### Water Activity

The water activity (Aw) was evaluated in fresh samples using the Aqualab 4TE Decagon^®^ equipment (Meter Group, Pullman, WA, United States) which analyzes the dew point in a cooled mirror. The vapor pressure point of the fresh sample comes into equilibrium with the air in a closed chamber that contains a mirror. Detection is a result of water condensing on the mirror. Once in equilibrium, the relative humidity of the air in the chamber is equal to the water activity in the sample.

#### Fixed Mineral Residue

Fixed mineral residue (ash) determination was carried out according to AOAC method 930.05 (Association of Agricultural Chemists [AOAC], 2005). Aluminum crucibles previously tared and weighed containing 5 g of sample were placed in a muffle oven at 550°C for 6 h, until complete incineration. After that, the crucibles were cooled in a desiccator, weighed in an analytical balance, and the fixed mineral residue content was determined.

#### Total Proteins

Total protein analysis was performed according to the Kjeldahl technique described by the AOAC method 978.04 (Association of Agricultural Chemists [AOAC], 2005), with some adjustments. A 5 g portion of the lyophilized samples was added in a Kjeldahl digester tube followed by 2.5 g of copper sulfate and sodium sulfate (1:10) and 20 ml of sulfuric acid. The blank tube did not contain a freeze-dried sweet potato sample. The solution was heated at 180°C for 2 h and up to 400°C for about 3 h until complete digestion. The distillation took place with 25 ml of 50% NaOH, and the distilled product was collected with an Erlenmeyer flask containing 50 ml of 4% boric acid solution. The titration was performed with 0.1 N hydrochloric acid, and total proteins were determined.

#### Total Lipids

The determination of total lipids with Soxhlet equipment was performed with modifications by the AOAC method 930.9 (Association of Agricultural Chemists [AOAC], 2005) with modifications. A 5 g of lyophilized sample was placed on filter paper tied with degreased wool yarn, transferred to a cellulose cartridge, and stored in the equipment’s glassware. A flat-bottomed flask was preheated for 60 min at 105°C, cooled in a desiccator, and tared. Then, 160 ml of Hexane was added to the flask. The balloon was connected to the equipment and heated for 6 h for lipid extraction. Afterward, the flat-bottomed flasks containing the lipids were cooled in a desiccator and weighted with an analytical balance. Total lipids were determined according to the method.

#### Total Carbohydrates

The evaluation of total carbohydrates content in the samples was performed by the difference between 100 (total percentage of substances present in the sample) and the sum of the content fractions obtained for the parameters: moisture, ash, total proteins, and total lipids.

### Total Dietary Fiber

Total fiber analysis was performed using an enzyme kit provided by Megazyme ^®^ (Megazime, Wicklow, Ireland), according to the 32-05.01 method described by the American Association of Cereal Chemists (American Association of Cereal Chemists [AACC], 2010), with some adjustments. Briefly, 1 g samples were carefully weighed into falcon tubes, with the addition of 50 μl of 0.08 M phosphate buffer (pH 6), 50 μl of α-Amylase [Enzyme Commission Number (EC), 3.2.1.1], and placed in a water bath at 100°C for 15 min with continuous shaking. The tubes were removed and cooled in an ice bath followed by the addition of 100 μl of Protease (EC, 3.4.21.62) and placed again in a hot bath at 60°C for 30 min with constant agitation. Then, 200 μl of amyloglucosidase (EC 3.2.1.3) was added, followed by a 60°C water bath with stirring. In each sample, 225 ml of 95% ethanol preheated at 60°C were added, followed by 60 min of rest. Filtration took place in a 40/100 micro-Gooch crucible containing 0.1 mg of celite placed on an Erlenmeyer flask connected to a vacuum system. The beaker was washed with 78% ethanol, and two portions of 95% ethanol and acetone were added to the Gooch crucible residues. The crucibles were kept overnight in an oven at 103°C. One replicate (R1) was followed for protein analysis by the Kjeldahl method, and another replicate (R2) was followed for ash determination in a muffle oven at 525°C. Each analysis had a blank control without the sample. The total dietary fiber content was obtained by equation 3, and the blank value was measured by equation 4.


(3) $$T\,o\,t\,a\,l\,d\,i\,e\,t\,a\,r\,y\,f\,i\,b\,e\,r=\frac{\left(\frac{R\,1+R\,2}{2}\right)-p-A-B}{\frac{m\,1+m\,2}{2}}\,x\,100$$

Where: m1 = replicate weight 1; m2 = double weight 2; R1 = residue weight of replicate m1 (protein analysis); R2 = residue weight of the replicate m2 (ash analysis); A = ash weight of R2; p = weight of R1 proteins; B = blank value (equation 4);


(4) $$B=\frac{B\,R\,1+B\,R\,2}{2}-B\,P-B\,A$$

Where: BR = white residue; BP = protein replicate blank (BR1); BA = white ash replicates (BR2).

### Statistical Analysis

The data collected in this work were statistically analyzed employing a licensed TIBCO Statistica ^®^ Ultimate Academic version 14 software (Statsoft Inc, 2021) (TIBCO Software, Palo Alto, United States) and the free software RStudio version 3.6 “Planting of a Tree”’ (Rstudio Team, 2019) (RStudio, Massachusetts, United States). All analytical results were initially estimated by normality and homoscedasticity tests. The parametric data set was submitted to ANOVA and Tukey test (*p* < 0.05), where the mean and *SD* were used as estimators. Non-parametric data were evaluated by Kruskal–Wallis and Nemeny’s tests (*p* < 0.05), where the median and interquartile range were used as estimators. The sample screening was performed by the exploratory statistical methods factor analysis (FA) and principal component analysis (PCA).

Initially, the input dataset was standardized to match the conjecture related to the orthogonal factor model calculated by the correlation matrix. The standardization was done to mitigate the significant variability intrinsic to the raw results data, so each independent variable holds a variance equal to 1. Equation 5 was used for data standardization.


(5) $$Z=\frac{X-\bar{X}}{D\,P}$$

Where: X = number to be standardized; X = mean of values; SD = standard deviation.

The standardized data were rotated using the Varimax raw method (Kaiser, 1958). The data rotation has the function of identifying the maximum collinearity between the input variables to obtain adequate patterns with high correlation for the factors (Forina et al., 2014). The FA was employed to reproduce the original variability embodied in the independent variable resulting in fewer random variables called factors. The factors estimated are linked to the initial input data variance by calculating linear models (Cruz and Topa, 2009; Alkarkhi and Alqaraghuli, 2019).

The FA was performed on the set of results acquired for the 16 independent variables that described the characteristics of the sweet potato cultivars and new varieties. All the 16 independent variables that feed the first FA are named: Weight (We), Length (Le), Width (Wi), Water activity (Wa), Moisture (M), Energy value (Ev), Total proteins (P), Total lipids (L), Total carbohydrates (Carb), Total dietary fiber (Tdf), Ashes (Ash), L*, a*, b*, C, and h°. The independent variables will be aggregated into new interrelated variable subsets and combined using grouping factors.

The estimation of the factor number for the exploratory FA was performed from the eigenvalues that have a value >1, according to the Kaiser criterion (Kaiser, 1958). The purpose is to preserve in the system the new dimensions that can reproduce the continuous variance information in the original independent variables. Thus, 5 factors were selected with eigenvalues of 3.83, 3.27, 2.46, 2.41, and 1.26.

The PCA by the correlation matrix was applied to evaluate the main independent variables as a function of the calculated factors. The method aims to throw light on the data variance and covariance structure through linear combinations. The linear combinations generated by the exploratory statistical test are described as principal components, and do not correlate with each other (Armanino et al., 1987; Forina et al., 2014).

## Results

### Registered Cultivars and New Varieties Sweet Potatoes Morphology

The morphological analysis revealed that the weight and length of the tubers varied significantly (*p* < 0.05) among all samples (Table 2). The highest weight result was observed for SCS372 Marina with 443.82 g (329.25), and 17117 426.55 g (85.82). The values for the samples 17025-13 [331.97 g (116.54)], 17052 [325.79 g (230.34)], 17092-9 [386.13 g (300.11)], 17105-20 [388.87 g (438.91)], 17107-18 [417.89 g (443.63)], 17125-10 [415.8 g (209.14)], and 17162 [377.57 g (221.89)] were statistically similar (*p* < 0.05). The SCS367 Favorita [83.30 g (22.01)] has the lowest weight. The dissimilarity between SCS372 Marina and SCS367 Favorita was 81.23%.

**TABLE 2:** Morphological comparison between registered and new sweet potatoes varieties.

Sample	Weight $\tilde{\text{x}}$(IQR)	Length $\bar{\text{x}}$± SD	Width $\bar{\text{x}}$± SD	Visual pulp color	Morphological traits
SCS367 Favorita	83.30 (22.01)	12.43 ± 3.27^c^	2.81 ± 0.70^b^	Yellow	Small, long, elliptical to round; surface with few constrictions; primary-skin: light brown; secondary-skin: light orange.
SCS368 Ituporanga	307.90 (224.39)*	16.30 ± 2.39^bc^	5.02 ± 1.10^a^	Yellow	Large, oblong; smooth surface; primary-skin: yellow; secondary-skin: light yellow.
SCS369 Águas Negras	302.81 (182.88)*	18.70 ± 4.89^ab^	4.63 ± 1.30^a^	White	Large, obovate to elliptical; smooth surface; primary-skin: red; secondary-skin: cream.
SCS370 Luiza	246.49 (86.91)*^‡^	16.20 ± 3.43^bc^	4.66 ± 0.92^a^	Purple	Medium to small, round-elliptical; smooth surface; primary-skin: purple; secondary-skin: purple.
SCS371 Katiy	306.64 (156.47)*^‡^	19.95 ± 5.01^ab^	3.93 ± 0.65^ab^	White	Large, long elliptical; surface with slight horizontal constrictions; primary-skin: brown; secondary-skin: cream.
SCS372 Marina	443.82 (329.25)*	20.50 ± 4.63^ab^	5.06 ± 1.01^a^	Yellowish/orange	Large, round elliptical; smooth surface; primary-skin: light brown; secondary-skin: orange.
Darci	314.72 (392.87)*	24.19 ± 2.68^a^	4.69 ± 1.58^a^	White	Large, elliptical to oblong; surface with slight longitudinal grooves; primary-skin: light purple; secondary-skin: cream.
Leandro	265.68 (146.06)*	21.63 ± 4.87^ab^	3.65 ± 0.31^ab^	White	Large, elliptical; surface with small veins; primary-skin: light brown; secondary-skin: cream.
17007-15	263.74 (82.03)*^‡^	19.56 ± 1.80^ab^	4.42 ± 0.97^a^	Orange	Large, elliptical; surface with veins and constrictions; primary skin: brown; secondary-skin: orange.
17025-13	331.97 (116.54)*	16.20 ± 3.63^bc^	5.04 ± 1.23^a^	Orange	Large, round; surface with slight constrictions; primary-skin: purple; secondary-skin: cream.
17052	325.79 (230.34)*	18.89 ± 3.69^ab^	4.87 ± 0.85^a^	Yellow	Medium, round elliptical; surface with slight horizontal constrictions; primary-skin: orange; secondary-skin: light orange.
17082-8	314.93 (264.44)*	22.08 ± 4.93^ab^	4.68 ± 0.81^a^	Yellow	Large, long elliptical; smooth surface; primary-skin: light orange; secondary-skin: cream.
17092-9	386.13 (300.11)*	21.00 ± 2.81^ab^	4.49 ± 0.81^a^	White	Large, long oblong; surface with light veins; primary-skin: light brown; secondary-skin: cream.
17105-20	388.87 (438.91)*	20.84 ± 5.59^ab^	5.22 ± 1.37^a^	Light Orange	Large, long oblong; veined surface; primary-skin: light brown; secondary-skin: cream.
17107-18	417.89 (443.63)**	21.78 ± 5.32^ab^	4.84 ± 0.90^a^	Light Orange	Large, long oblong; smooth surface; primary-skin: light purple; secondary-skin: light orange.
17117	426.55 (178.39)*	22.97 ± 4.71^a^	4.53 ± 1.04^a^	Orange	Large, long oblong; surface with slight vertical and horizontal constrictions; primary-skin: brown; secondary-skin: orange.
17125-10	415.80 (209.14)*	20.33 ± 3.30^ab^	4.65 ± 1.45^a^	White/yellowish	Large, round to round elliptical; surface with slight vertical constrictions; primary-skin: brown; secondary-skin: cream.
17162	377.57 (221.89)*	22.03 ± 5.71^ab^	4.82 ± 0.62^a^	Yellow	Large, long oblong to irregular elliptical; surface with slight veins and constrictions; primary-skin: light purple; secondary-skin: cream.

*Different symbols in the weight column show significant differences between samples with 95% certainty by Kruskal–Wallis/Nemenyi tests. Different letters in length and width columns show significant differences between samples with 95% certainty by ANOVA/Tukey. $\tilde{\text{x}}$(IQR), median and Interquartile Range. $\bar{\text{x}}$± SD, mean and standard deviation.*

The samples Darci (24.19 cm ± 2.68) and 17117 (22.97 cm ± 4.71) exhibited higher length, along with SCS369 Águas Negras (18.7 cm ± 4.89), SCS371 Katiy (19.95 cm ± 5.01), SCS372 Marina (20.50 cm ± 4.63), and the new varieties Leandro (21.63 cm ± 4.87), 17007-15 (19.56 cm ± 1.80), 17052 (18.89 cm ± 3.69), 17082-8 (22.08 cm ± 4.93), 17092-9 (21 cm ± 2.81), 17105-20 (20.84 cm ± 5.59), 17107-18 (21.78 cm ± 5.32), 17125-10 (20.33 cm ± 3.30), and 17162 (22.03 cm ± 5.71), which showed no significant difference (*p* < 0.05) for that physical variable. The cultivars SCS367 Favorita (12.43 cm ± 3.27), SCS368 Ituporanga (16.3 cm ± 2.39), and SC370 Luiza (16.2 cm ± 3.43) showed statistically similar results with the shortest lengths. The length difference between Darci and SCS367 Favorita was 48.61%.

However, width did not show significant variation among the samples. The new variety 17105-20 showed the highest result of 5.22 cm ± 1.37, which was significantly similar (*p* < 0.05) to SCS372 Marina (5.06 cm ± 1.01), 17025-13 (5.04 cm ± 1.23), SCS368 Ituporanga (5.02 cm ± 1.10), SCS369 Águas Negras (4.63 cm ± 1.30), SCS370 Luiza (4.66 cm ± 0.92), and the new varieties Darci (4.69 cm ± 1.58), 17007-15 (4.42 cm ± .97), 17052 (4.87 cm ± 0.85), 17082-8 (4.68 cm ± 0.81), 17092-9 (4.49 cm ± 0.81), 17105-20 (5.22 cm ± 1.37), 17107-18 (4.84 cm ± 0.9), 17117 (4.53 cm ± 1.04), 17125-10 (4.65 cm ± 1.45), and 17162 (4.82 cm ± 0.62). The SCS367 Favorita (2.81 cm ± 0.7) and Leandro (3.65 cm ± 0.31) exhibited the lowest thickness values and were statistically similar. Thickness results had a 46.16% of variation between highest and lowest value.

Overall, morphological characteristics of the samples (Table 2) were appraised as large, with round shape, elliptical oblong to elliptical and large thickness. Skin visual color was mostly light brown for the superficial skin and cream for the secondary skin. The root surfaces were predominantly irregular, ranging from smooth samples to samples with slight constrictions and protruding veins.

### Pulp Instrumental Color

Table 3 shows the results obtained from the pulp fraction instrumental color of all the samples analyzed. The set color of pulp samples exhibited remarkable differences, but the color scope ranges between white, yellow, orange, and purple.

**TABLE 3:** Instrumental pulp color of registered and new varieties of sweet potato cultivars.

Samples	L* $\tilde{\text{x}}$(IQR)	a* $\tilde{\text{x}}$(IQR)	b* $\tilde{\text{x}}$(IQR)	C $\tilde{\text{x}}$(IQR)	h° $\tilde{\text{x}}$(IQR)
SCS367 Favorita	71.63 (1.36)	19.59 (0.89)	47.31 (1.88)*^†^	51.21 (1.40)*^‡^	67.52 (1.75)
SCS368 Ituporanga	83.54 (1.36)	−3.98 (0.34)	31.61 (0.66)	31.86 (0.70)	97.23 (0.11)
SCS369 Águas Negras	84.10 (1.45)	−3.04 (0.53)	24.08 (0.73)	24.27 (0.79)^‡^	97.24 (1.06)
SCS370 Luiza	27.90 (1.83)^‡^	30.83 (1.42)*	−8.42 (2.28)^‡^	31.96 (1.93)	344.74 (3.48)*
SCS371 Katiy	81.79 (3.05)	−2.50 (0.38)	24.78 (2.66)	24.91 (2.60)^‡^	95.83 (1.56)
SCS372 Marina	79.14 (1.21)	3.53 (1.18)	42.70 (0.87)	42.88 (0.81)	85.23 (1.56)
Darci	83.40 (2.07)	−4.08 (0.80)	23.30 (0.77)^‡†^	23.54 (0.77)^‡^	100.03 (1.82)
Leandro	82.90 (1.15)	−3.56 (0.35)	28.86 (0.92)	29.08 (0.95)	97.26 (0.56)
17007-15	57.37 (0.90)^‡^	32.28 (2.01)*	48.21 (0.87)*	58.02 (1.82)*	56.51 (1.46)’^†^
17025-13	71.04 (3.31)	24.15 (1.29)	43.39 (1.80)	49.66 (0.96)	60.91 (2.28)
17052	83.89 (2.18)	−3.47 (1.41)	41.57 (1.42)	41.63 (1.39)	94.64 (1.92)
17082-8	82.80 (0.87)	−0.75 (0.15)	42.80 (0.78)	42.81 (0.78)	91.76 (0.70)
17092-9	85.19 (0.79)*	−4.05 (0.32)	24.03 (0.23)	24.37 (0.28)^‡^	99.69 (0.63)*^‡^
17105-20	72.55 (1.26)	14.15 (1.81)	39.79 (0.28)	42.15 (0.80)	70.46 (2.17)
17107-18	74.57 (2.58)	17.66 (4.07)	41.11 (2.77)	44.74 (4.30)	66.18 (3.29)
17117	65.46 (0.35)	24.93 (1.13)	40.82 (1.07)	47.95 (1.38)	59.04 (0.93)’^‡^
17125-10	83.81 (2.90)	−3.71 (1.88)	42.45 (1.58)	42.54 (1.48)	95.53 (2.69)
17162	84.94 (2.85)	−5.30 (1.26)^‡^	31.75 (1.35)	32.01 (1.32)	99.34 (2.47)

*Different symbols in a column show significant differences between samples with 95% certainty by Kruskal–Wallis and Nemenyi tests. $\tilde{\text{x}}$(IQR), median and interquartile range.*

The new variety 17092-9 showed the highest result for luminosity with a L* 85.19 (0.79), which was significantly different (*p* < 0.05) from the lowest values of samples SCS370 Luiza 27.9 (1.83) and 17007-15 57.37 (0.9). There was a statistical similarity for the instrumental color a* parameter values between 17007-15 [32.28 (2.01)] and SCS370 Luiza [30.83 (1.42)]. The lowest value recorded for a* was 17162 [−5.3 (1.26)]. Furthermore, the parameter b* 17007-15 [48.21 (0.87)] exhibited the highest result which was statistically similar only to SCS367 Favorita [47.31 (1.88)] which in turn was statistically similar to Darci [23.3 (0.77)]. Darci and SCS370 Luiza [−8.42 (1.42)] were significantly similar (*p* < 0.05).

Saturation parameter C* was highest for 17005-15 [58.02 (1.82)] and SCS367 Favorita [51.21 (1.4)] which were statistically similar. In addition, SCS367 Favorita was similar to SCS371 Katiy [24.91 (2.6)], 17092-9 [24.37 (0.28)], SCS369 Águas Negras [24.27 (0.79)], and Darci [23.54 (0.77)]. On the other hand, SCS370 Luiza [344.74 (3.48)] had the highest h° value statistically similar (*p* < 0.05) to 17092-9 [99.69 (0.63)], which in turn was similar to 17117 [59.04 (0.93)]. The lowest value for this parameter was for 17007-15 [56.51 (1.46)], which did not show a significant difference for 17117.

### Proximal Composition and Total Dietary Fiber

Sweet potato samples showed a significant difference (*p* < 0.05) for all independent variables as illustrated in Table 4. The water activity was close to 1 for all samples. Samples SCS370 Luiza (0.99 ± 0.01), SCS368 Ituporanga (0.99 ± 0.01), SCS371 Katiy (0.99 ± 0.01), SCS372 Marina (0.99 ± 0.01), 17007-15 (0.99 ± 0.01), 17052 (0.99 ± 0.01), 17092-9 (0.99 ± 0.01), 17117 (0.99 ± 0.01), and 17125-10 (0.99 ± 0.01) exhibited the highest values, which did not differ statistically (*p* < 0.05) from SCS367 Favorita (0.98 ± 0.01), SCS369 Águas Negras (0.98 ± 0.01), Leandro (0.98 ± 0.01), 17025-13 (0.98 ± 0.01), and 17082-8 (0.98 ± 0.01). In contrast, the sweet potato new variety named 17162 (0.97 ± 0.01) exhibited the lowest value for water activity.

**TABLE 4:** Proximal composition and total dietary fiber content in registered and new varieties of sweet potato cultivars.

Sample	Water activity	Moisture (%)	Energy value	Total proteins	Total lipids	Total	Total dietary	Ashes
	$\bar{\text{x}}$± *SD*	$\bar{\text{x}}$± *SD*	(kcal.100 g^–1^)	(g.100g^–1^ DM)	(g.100g^–1^ DM)	carbohydrates	fiber	(g.100g^–1^ DM)
			$\bar{\text{x}}$± *SD*	$\bar{\text{x}}$± *SD*	$\bar{\text{x}}$± *SD*	(g.100g^–1^ DM)	(g.100g^–1^ DM)	$\bar{\text{x}}$± *SD*
						$\bar{\text{x}}$± *SD*	$\bar{\text{x}}$± *SD*	
SCS367 Favorita	0.98 ± 0.01^abcd^	81.64 ± 1.21^abcde^	67.42 ± 5.13^cde^	2.60 ± 0.10^hij^	1.01 ± 0.45^abc^	11.98 ± 1.20^def^	4.45 ± 0.86^b^	2.77 ± 0.17^bcde^
SCS368 Ituporanga	0.99 ± 0.01^abcd^	80.16 ± 0.61^abcdefg^	78.16 ± 1.84^abcd^	2.24 ± 0.22^j^	1.77 ± 0.14^a^	13.30 ± 0.56^cd^	6.58 ± 1.62^ab^	2.52 ± 0.13^cde^
SCS369 Águas Negras	0.98 ± 0.01^abcd^	83.13 ± 0.70^ab^	66.14 ± 5.27^de^	4.91 ± 0.11^bc^	0.95 ± 0.42^abc^	9.47 ± 0.44^efg^	5.62 ± 1.62^b^	1.53 ± 0.14^h^
SCS370 Luiza	0.99 ± 0.01^a^	79.68 ± 0.80^bcdefgh^	75.32 ± 1.79^abcde^	3.60 ± 0.09^ef^	1.07 ± 0.52^abc^	12.81 ± 1.02^de^	7.40 ± 1.89^ab^	2.83 ± 0.15^bcd^
SCS371 Katiy	0.99 ± 0.01^abcd^	80.77 ± 0.68^abcdef^	75.78 ± 4.97^abcde^	3.12 ± 0.10^fghi^	0.99 ± 0.54^abc^	13.61 ± 0.23^cd^	8.08 ± 1.98^ab^	1.51 ± 0.45^h^
SCS372 Marina	0.99 ± 0.01^abcd^	76.92 ± 1.43^gh^	87.78 ± 4.79^a^	1.45 ± 0.16^k^	0.92 ± 0.23^abc^	18.42 ± 1.52^a^	12.60 ± 1.26^a^	2.28 ± 0.13^def^
Darci	0.98 ± 0.01^cd^	80.55 ± 0.04^abcdef^	70.30 ± 0.41^bcde^	3.87 ± 0.15^de^	1.00 ± 0.03^abc^	11.45 ± 0.30^defg^	4.44 ± 1.57^b^	3.13 ± 0.11^b^
Leandro	0.98 ± 0.01^abcd^	82.41 ± 1.62^abcd^	68.81 ± 8.84^bcde^	5.22 ± 0.10^b^	1.09 ± 0.52^abc^	9.53 ± 0.92^efg^	4.78 ± 1.39^b^	1.73 ± 0.08^fgh^
17007-15	0.99 ± 0.01^abcd^	79.32 ± 0.88^defgh^	80.17 ± 4.47^abcd^	7.35 ± 0.12^a^	1.71 ± 0.08^ab^	8.85 ± 1.06^fg^	7.67 ± 1.91^ab^	3.77 ± 0.25^a^
17025-13	0.98 ± 0.01^abcd^	76.28 ± 0.86^h^	88.43 ± 3.65^a^	2.57 ± 0.44^ij^	0.95 ± 0.15^abc^	17.38 ± 0.45^ab^	10.59 ± 2.82^ab^	2.81 ± 0.08^bcd^
17052	0.99 ± 0.01^abc^	81.33 ± 0.89^abcde^	71.69 ± 2.77^bcde^	7.04 ± 0.15^a^	1.24 ± 0.26^abc^	8.08 ± 1.11^g^	4.77 ± 1.76^b^	2.30 ± 0.03^def^
17082-8	0.98 ± 0.01^abcd^	79.58 ± 1.00^cdefgh^	74.89 ± 4.26^abcde^	4.43 ± 0.20^cd^	0.89 ± 0.13^abc^	12.28 ± 1.38^def^	9.70 ± 1.24^ab^	2.81 ± 0.35^bcd^
17092-9	0.99 ± 0.01^abcd^	78.48 ± 0.50^efgh^	81.60 ± 1.88^abc^	3.18 ± 0.14^fgh^	1.09 ± 0.09^abc^	14.76 ± 0.77^bcd^	8.10 ± 1.89^ab^	2.49 ± 0.26^cde^
17105-20	0.98 ± 0.01^bcd^	80.15 ± 1.00^abcdefg^	71.23 ± 3.72^bcde^	2.90 ± 0.15^ghi^	0.97 ± 0.08^abc^	12.72 ± 0.88^de^	6.48 ± 1.59^ab^	3.26 ± 0.07^ab^
17107-18	0.98 ± 0.01^abcd^	77.75 ± 2.23^fgh^	83.48 ± 9.23^ab^	2.70 ± 0.40^hij^	0.66 ± 0.04^c^	16.68 ± 2.56^abc^	10.20 ± 1.26^ab^	2.20 ± 0.03^efg^
17117	0.99 ± 0.01^abcd^	82.96 ± 1.24^abc^	62.46 ± 4.43^e^	3.31 ± 0.17^efg^	1.22 ± 0.23^abc^	9.55 ± 1.44^efg^	7.00 ± 2.06^ab^	2.95 ± 0.05^bc^
17125-10	0.99 ± 0.01^ab^	81.21 ± 0.45^abcde^	74.46 ± 1.54^abcde^	2.11 ± 0.15^j^	1.19 ± 0.28^abc^	13.82 ± 0.72^bcd^	6.85 ± 1.76^ab^	1.65 ± 0.10^gh^
17162	0.97 ± 0.01^d^	83.32 ± 1.90^a^	60.96 ± 8.06^e^	4.72 ± 0.17^bc^	0.82 ± 0.15^bc^	8.67 ± 2.08^fg^	4.66 ± 1.39^b^	2.46 ± 0.23^cde^

*Different letters in the same column show significant differences between samples with 95% certainty by ANOVA and Tukey tests. DM, dry matter. $\bar{\text{x}}$± SD, mean and standard deviation.*

Moisture content showed variations among all the samples. Sample 17162 had the highest percentage (83.32 ± 1.9) and was statistically similar to samples SCS369 Águas Negras (83.13 ± 0.7), 17117 (82.96 ± 1.24), Leandro (82.41 ± 1.62), SCS367 Favorita (81.64 ± 1.21), 17052 (81.33 ± 0.89), 17125-10 (81.21 ± 0.45), SCS371 Katiy (80.77 ± 0.68), Darci (80.55 ± 0.04), SCS368 Ituporanga (80.16 ± 0.61), and 17105-20 (80.15 ± 1). The lowest results for moisture content were recorded for 17025-13 (76.28 ± 0.86), statistically similar (*p* < 0.05) to SCS372 Marina (76.92 ± 1.43), 17107-18 (77.75 ± 2.23), 17092-9 (78.48 ± 0.5), 17007-15 (79.32 ± 0.88), 17082-8 (79.58 ± 1), and SCS370 Luiza (79.68 ± 0.8). The difference between the highest and lowest moistures was 8.45%.

The proximal composition data of the sweet potato cultivars and new varieties enable estimation of the total energy value in kilocalories (Kcal), according to the Atwater conversion values (Nichols, 1994; Welsh, 1994). Variety 17025-13 (88.43 ± 3.65) had the highest variable energy and was statistically similar to SCS372 Marina (87.78 ± 4.79), 17107-18 (83.48 ± 9.23), 17092-9 (81.6 ± 1.88), 17007-15 (80.17 ± 4.47), SCS368 Ituporanga (78.16 ± 1.84), SCS371 Katiy (75.78 ± 4.97), SCS370 Luiza (75.32 ± 1.79), 17082-8 (74.89 ± 4.26), and 17125-10 (74.46 ± 1.54). In contrast, the new variety 17162 (60.96 ± 8.06) exhibited the lowest energy, followed by 17117 (62.46 ± 4.43), SCS369 Águas Negras (66.14 ± 5.27), SCS368 Favorita (67.42 ± 5.13), Leandro (68.81 ± 8.84), Darci (70.3 ± 0.41), 17105-20 (71.23 ± 3.72), 17052 (71.69 ± 2.77), and also 17125-10, 17082-8, SCS370 Luiza and SCS371 Katiy.

The results for total protein (on a dry basis) revealed that samples 17007-15 (7.35 ± 0.12) and 17052 (7.04 ± 0.15) had the highest content. The lowest value was observed in SCS372 Marina (1.45 ± 0.16). For total lipids, all sweet potatoes had a low content, which is characteristic of tuberous roots, so SCS368 Ituporanga (1.77 ± 0.14) stood out from the others and was significantly different (*p* < 0.05) from 17162 (0.82 ± 0.15) and 17107-18 (0.66 ± 0.04).

The variant SCS372 Marina (18.42 ± 1.52) exhibited the highest content for total carbohydrates and was statistically similar to 17025-13 (17.38 ± 0.45) and 17107-18 (16.68 ± 2.56). The lowest values for total carbohydrates belonged to 17052 (8.08 ± 1.11), 17162 (8.67 ± 2.08), 17007-15 (8.85 ± 1.06), SCS369 Águas Negras (9.47 ± 0.44), Leandro (9.53 ± 0.92), 17117 (9.55 ± 1.44), and Darci (11.45 ± 0.3) without any significant differences.

The results for total dietary fiber revealed that cultivar SCS372 Marina (12.6 ± 1.26) had the highest value, followed by samples 17025-13 (10.59 ± 2.82), 17107-18 (10.2 ± 1.26), 17082-8 (9.7 ± 1.24), 17092-9 (8.1 ± 1.89), SCS371 Katiy (8.08 ± 1.98), 17007-15 (7.67 ± 1.91), SCS370 Luiza (7.4 ± 1.89), 17117 (7 ± 2.06), 17125-10 (6.85 ± 1.76), SCS369 Ituporanga (6.58 ± 1.62), and 17105-20 (6.48 ± 1.59), which did not show statistical differences (*p* < 0.05) among themselves. Samples Darci (4.44 ± 1.57), SCS368 Favorita (4.45 ± 0.86), 17162 (4.66 ± 1.39), 17052 (4.77 ± 1.76), Leandro (4.78 ± 1.39) and SCS369 Águas Negras (5.62 ± 1.62) had the lowest values.

Ash content was the highest for the new variety 17007-15 (3.77 ± 0.25), followed by 17105-20 (3.26 ± 0.07), both similar to each other according to statistical analysis. The cultivar SCS371 Katiy (1.51 ± 0.45) exhibited the lowest ash content, together with SCS369 Águas Negras (1.53 ± 0.14), 17125-10 (1.65 ± 0.1), and Leandro (1.74 ± 0.08).

### Factor Analysis and Principal Component Analysis

The morphological, colorimetric, and physicochemical analyses carried out for the cultivars and new varieties of sweet potatoes aimed to elucidate their physical structure and composition. The variance observed in the results was pointed out by statistical inference methods and revealed intrinsic differences in samples as the independent variable. However, for sample screening, other statistical methods had to be applied to the data. So, the results were subjected to the FA exploratory statistical analysis. Supplementary Table 1 presents the correlations between independent variables.

The Supplementary Table 1 numerical value analysis suggests the clustering of the 16 independent input variables into two groups, the first group formed by We, Le, and Wi, which are related to the morphology of the samples. The other group was formed by Wa, M, Ev, P, L, Carb, Tdf, Ash, L*, a*, b*, C*, and h°, which describes the proximal composition, total dietary fiber content, and instrumental pulp color. Thus, the sample correlation matrix eigenvalues were obtained. The eigenvalue represents the importance that each new factor has concerning the explanatory variability of the data set. The eigenvalues results are presented in Supplementary Table 2.

It is possible to assess by Supplementary Table 2 that the 5 factors combined explained 82.64% of the total variation intrinsic to the input data. Factor 1 explained 23.93% of the data variability, while Factor 2 explained 20.42%, Factor 3: 15.35%, Factor 4: 15.04%, and Factor 5: 7.91%. The Supplementary Table 3 displays the relationship between the response variables obtained during the analyses and the 5 factors calculated by FA.

According to Supplementary Table 3, the factor loadings correlation between the 5 factors and the 16 input independent variables were observed. The M, Ev, Carb, and Tdf had a high relationship with Factor 1 (23.93% of the variability) and they correlate with each other on this factor. The L* and a* are related to each other and correlated with Factor 2 (20.42% of the variability). Variables b* and h° had a loading value related to Factor 3 (15.35% of variability). The Wa and ash were correlated and linked to Factor 4 (15.04% of the variability). The We and Le morphology variables have a loading value linked to Factor 5, with 7.91% of the total explanatory variance.

The Supplementary Figure 2 shows the Scree-plot with eigenvalues obtained for every 16 independent variables and confirms the adoption of the 5 factors for the first exploratory FA (Kaiser, 1958). The Supplementary Table 4 presents the independent variable communality values with relation to the new factors. According to the Kaiser–Merkin–Olkin (KMO) adjustment criterion (Kaiser, 1970), the communality value for each variable concerning the last factor (Factor 5) must be >0.7. Due to the lower load observed for Width (0.65), P (0.42), and L (0.68), a second exploratory FA was performed to verify the adequacy of the input data.

**FIGURE 1:**
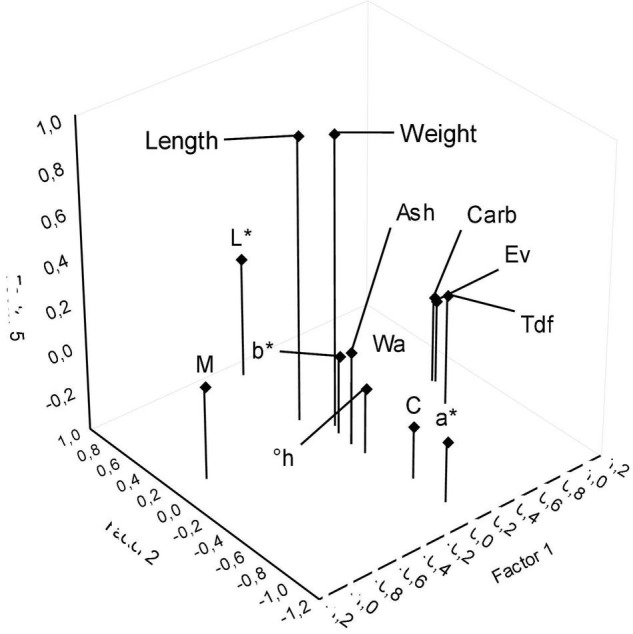
Second exploratory factor analysis: three-dimensional distribution of independent variables as a function of Factors 1, 2, and 5.

The second exploratory FA was performed with the suppression of the variables with the lowest communality from the input dataset (Width, P, and L). The first exploratory FA was able to identify 13 independent variables that summarize the preliminary information related to the original variability. Thus, a new exploratory FA was performed with only the 13 variables with great communality, namely: We, Le, Wa, M, Ev, Carb, Tdf, Ash, L*, a*, b*, C, and h°. Supplementary Table 5 shows the sample correlations obtained for the 13 independent variables of the second exploratory FA. Thus, 5 factors were used again in which each factor had an eigenvalue >1 (Kaiser, 1958). The factor eigenvalues of the second exploratory FA increased to 3.63, 2.99, 2.42, 1.87, and 1.05. Figure 1 presents the 13 input variables of the second FA, and the Supplementary Table 6 illustrates the results of the five factors cumulative percentage.

The PCA was performed for the independent variables with greater communality pointed by FA. The We, Le, Wa, M, Ev, Carb, Tdf, Ash, L*, a*, b*, C*, and h° variables were used as the random input vector. The raw data were previously standardized by equation 5 to obtain independent variables centered on zero, with an approximate variance of 1. The Supplementary Table 5 illustrates the correlations between the 13 independent variables, and Supplementary Table 9 presents the mean and standard deviation.

For the PCA test, a maximum number of 4 principal components (PC) capable of synthesizing and describing the explanatory variability of the input data was selected. The eigenvalues for the 4 PCs were 3.63, 2.99, 2.42, and 1.87. Supplementary Table 10 presents the eigenvalues, and Supplementary Table 11 illustrates the 4 PCs scores as a function of each 13 independent variables.

According to Supplementary Table 10, PC 1 had a total explanatory variance of 27.96%, while PC 2 had 23.03%, PC 3 18.60%, and PC 4 explained 14.42%. Supplementary Table 11 indicates that PC 1 can be interpreted as a dimension that associates the samples’ independent variables of nutritional quality. Considering the high scores for PC 1 (Supplementary Table 11), it is possible to affirm that M, Ev, Carb, Tdf had a higher positive correlation. The a* color parameter also tends to PC 1.

The PC 2 and PC 3 represent dimensions of comparison between independent variables related to the samples’ proximate composition and color analysis. The highlights estimated by the scores are due to the color parameters, notably the L* with the highest correlation for PC 2. In contrast, parameters b* and C were correlated to PC 3. But PC 4 stood out as being an index of the morphological quality of sweet potato tuberous roots. Clustered with the independent variables of morphology, there were also attributes related to proximate composition. The highlighted scores for PC 4 emphasize the independent variables We, Le, Wa, and Ash.

In conformity with Supplementary Table 11, it is possible to evaluate the weighting coefficients calculated between the independent variables and the PCA main components, which have numerically balanced values. This aspect is linked to the balanced distribution of total explanatory variance among the components. Thus, the principal explanation is focused on PC 1 and PC 4, which clustered independent variables of greater interest. Therefore, PC 1 and PC 4 can be interpreted as indexes that describe the nutritional quality and morphological aspects of the samples.

The Supplementary Table 9 confirms that the independent variables hold a variance of approximately 1, and there is no direct dominance of any specific variable. So, the influence source of the distinction between the coefficient values arrives from the correlation between the independent variables. The Supplementary Table 12 illustrates the 4 PC score results for the PCA as a function of the 18 sweet potato samples (Table 1).

The Supplementary Table 12 indicates the relationship between samples and the 4 PCs calculated by the test. The samples with the highest individual scores for a specific PC were directly correlated with the analytical results observed for the characterization of independent variables (Tables 2–4). A closer evaluation reveals that the independent variables M, Ev, Carb, Tdf, and a* tend toward PC 1, an index of sweet potato samples’ nutritional quality, and the scores of these variables for PC 1 (Supplementary Table 11) were −0.8, 0.75, 0.67, 0.77, and 0.59. So, the samples that had the highest results related to these specific variables are 17162, 17025-13, SCS372 Marina, SCS370 Luiza, and 17007-15 (Table 4), which hold a high score with PC 1 with results −3.47, 3.15, 3.88, 3.11, and −2.55, respectively (Supplementary Table 12).

Considering the PC 4 an index related to the morphology of the samples, the clustered variables were Le, We, Ma, and Ash, with scores −0.71, −0.53, −0.65, and −0.65 (Supplementary Table 11). Tables 2, 4 show significant results for sweet potato samples Darci, SCS372 Marina, and 17005-15, with scores 1.77, −1.33, and −2.23 (Supplementary Table 12), respectively.

The Supplementary Figure 4 illustrates the eigenvalue percentage of each 13 independent variable. Moreover, Supplementary Table 13 shows the variables’ communality values about the 4 PC. The Supplementary Table 13 evaluation confirms the adjustment obtained with the second exploratory FA (Supplementary Table 8), with the objective of grouping multicollinearity independent variables. The Supplementary Figure 4 confirms the quality of the model as a function of the principal components’ total explanatory variance percentage.

**FIGURE 2:**
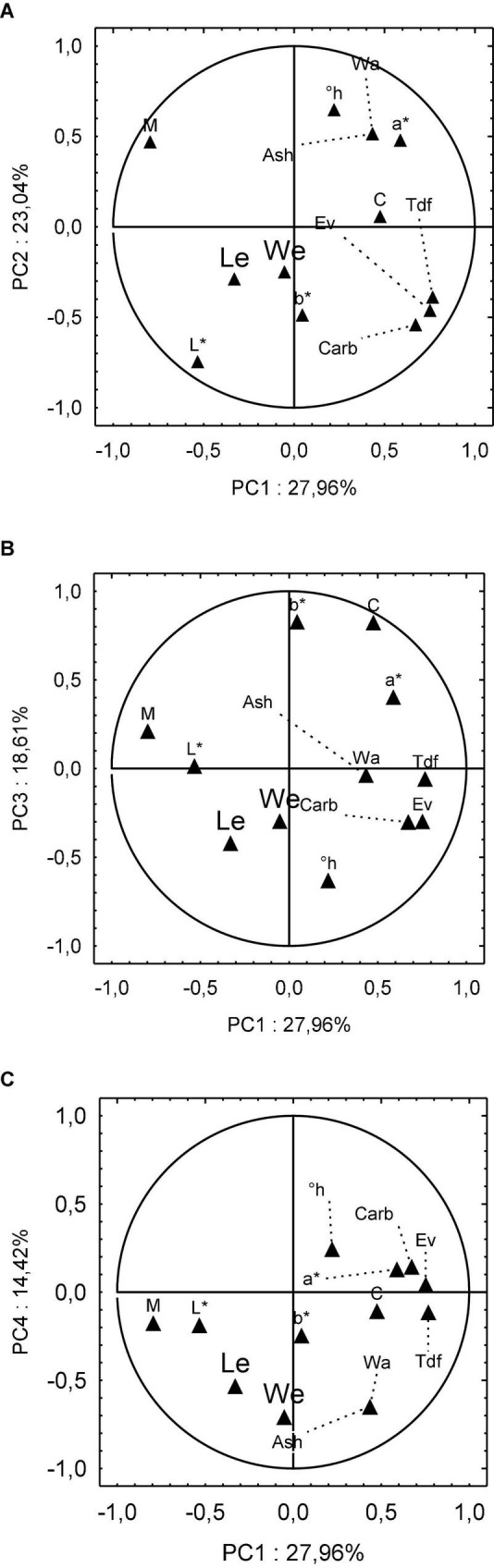
Principal component analysis (PCA) distribution of independent variables: **(A)** PC1 × PC2, **(B)** PC1 × PC3, and **(C)** PC1 × PC4.

Figure 2A shows the dispersion of independent variables as a function of PC 1 and PC 2, which together hold 51% of the data explanatory variability. Variables a*, Wa, Ash, h°, and C* are clustered in the upper right quadrant. Isolated in the upper left quadrant and tended to PC 2 is M. In the lower right quadrant are the variables Tdf, Ev, Carb, and b*, and in the lower left quadrant are the variables We, Le, and L*. It is possible to observe the formation of two critical clusters that explain the data. First, there is a cluster in the lower right quadrant concerning the nutritional quality of the samples that gather Tdf, Ev, and Carb. Independent variables We and Le are clustered in the lower left quadrant, representing the morphological quality of sweet potatoes. Both clusters have a low relationship with PC 2, but they are distinguished by the association with PC1, higher for the nutritional cluster and lower for the morphological cluster.

Figure 2B illustrates the independent variables’ dispersion according to PC 1 and PC 3. Again, the upper right quadrant groups the instrumental color parameters C*, b*, and a* in this set. In the upper left quadrant are the M. In the lower right quadrant are the variables Tdf, Ev, Carb, along with Wa, Ash, and h°, and in the lower left quadrant are the variables We and Le. Finally, on the abscissa axis is the variable L* with low adherence to PC 1 and null for PC 3. Moreover, Figure 2C shows a new dispersion between the variables, which in the upper right quadrant are Ev, Carb, a*, and h°. In the lower right quadrant are clustered Tdf, C, Wa, Ash, and b*, and in the lower left quadrant are the variables We, Le, L*, and M.

**FIGURE 3:**
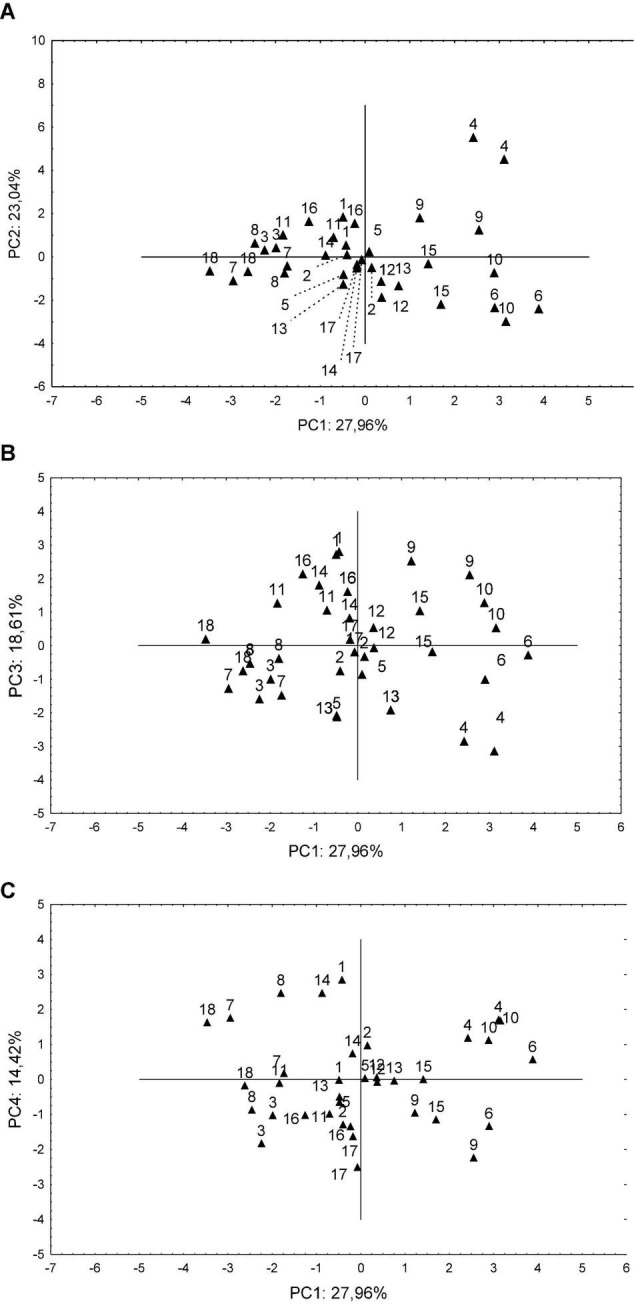
PCA Distribution of samples: **(A)** PC1 × PC2, **(B)** PC1 × PC3, and **(C)** PC1 × PC4. Note | Numbers are related to specific samples mentioned in Table 1.

The independent variable clustering observed in Figure 3 can be correlated with the dispersion of the dependent variables (samples), which is presented in Figure 3. Figure 3 is also correlated with Supplementary Table 12, especially with the arrangement of each sample in the scatter plot. Due to the size of sweet potato names, the graphic was composed of the sample numbers that were previously described in Table 1. Regarding the analysis of the PCA’s dispersion, the orientation was given to fully immersed samples in a specific quadrant.

Figure 3A congregates PC 1 and PC 2 (with 51% of the explanatory variability) and shows that these principal components resulted in a greater grouping of samples. In the upper right quadrant are samples SCS70 Luiza and 17007-15. In the upper left quadrant are samples SCS367 Favorita, SCS369 Águas Negras, 17052 and 17117. In the lower right quadrant, it is possible to observe the cluster of samples SCS372 Marina, 17025-13, 17107-18, and 17082-8, and in the lower-left quadrant is 17125-10, 17105-20, and 17162. The samples that were scattered between two quadrants are SCS368 Ituporanga, SCS371 Katiy, Darci, Leandro, and 17092-9.

Figure 3B is composed of PC 1 and PC 3 (46.57% of the explanatory variability). In the upper right quadrant with positive scores for both principal components (Supplementary Table 12) are 17025-13 and 17007-15. In the upper left quadrant, a positive score for PC 3 and a negative for PC 1 stands SCS367 Favorita, 17117, 17105-20, 17052. In the lower right quadrant are SCS372 Marina and SCS370 Luiza. In the lower left quadrant are SCS368 Ituporanga, Leandro, SCS369 Águas Negras and Darci. The samples that fall into more than one quadrant are SCS371 Katiy, 17082-8, 17092-9, 17107-18, 17125-10, and 17162.

Figure 3C shows PC 1 and PC 4 (42.38% of the explanatory variability). This PCA scatter plot is critical because it presents the dimensions that aggregate the nutritional quality (PC 1) and morphological quality (PC 4) samples index. Figure 3C, shows 17025-13 and SCS370 Luiza in the upper right quadrant. In the upper left quadrant are Darci and 17105-20. In the lower right quadrant are 17007-15, 17107-18. Thus, in the lower left quadrant are samples 17125-10, 17117, SCS369 Águas Negras and 17052. In this dimensional chart, the samples distributed in more than one quadrant are SCS367 Favorita, SCS368 Ituporanga, SCS371 Katiy, SCS372 Marina, Leandro, 17082- 8, 17092-9, and 17162.

## Discussion

### Morphological Aspects of Sweet Potatoes Samples

The tuberous root morphological characteristics varied within the samples and among the new varieties. Nonetheless, the mean results presented in Table 2 follow the morphological pattern that was observed in the sample crop. Sweet potato morphology variability may be linked to factors such as seasonality, crop farming management, climatic conditions, and natural plant characteristics (Karuri et al., 2010; Abdissa et al., 2012).

The independent variable weight is the main attribute for the rural producer’s decision to grow specific varieties of sweet potato as it is directly related to crop yield. Srinivas and Nedunchezhiyan (2020) cited variables that influenced Indian rural producers on the adoption of technologies capable of benefiting sweet potato production. The authors listed in their work several production technology variables that were associated with an opportunity for a possible increase in sweet potato agricultural productivity in different regions of India. An opinion survey revealed that the sweet potato varieties of high yield have a significant role in increasing productivity. The authors then developed a mathematical model to describe the main variables which are related to the increase in technology adoption, and plant variety increased acceptance quotient.

Mugumaarhahama et al. (2021) indicated that the incentive to use sweet potato varieties from improved plants is an alternative to increasing agricultural productivity. The work identified variables related to the decision to use improved crops by rural producers in the Democratic Republic of Congo. Certain factors such as schooling, income, the available area for cultivation, and labor may contribute to the increased adoption of improved sweet potato cultivars. The authors conclude that there is an urgency to stimulate higher sweet potato production in the evaluated regions and that the adoption of better cultivation practices in conjunction with improved sweet potato cultivars can contribute to this challenge. It is worth mentioning that the work is aligned with the African Union’s Agenda 2063 in terms of increasing productivity, sustainability, and food security, which highlights the importance of research on the topic.

Among the sweet potato samples evaluated in the study, cultivars SCS372 Marina and 17117 had the highest weight compared to the other roots. Samples 17025-13, 17052, 17092-9, 17105-20, 17107-18, 17125-10, and 17162 showed statistically similar results (Table 2). However, the cultivar SCS367 Favorita had the lowest weight, and its roots are naturally small. These data indicate that tuberous roots from the new variety of sweet potato plants had satisfactory weight compared to the registered cultivars.

Length results revealed that Darci and 17117 had the longest roots, and their values were similar to SCS369 Águas Negras, SCS371 Katiy, SCS372 Marina, Leandro, 17007-15, 17052, 17082-8, 17092-9, 17105-20, 17107-18, 17125-10, and 17162. The cultivars SCS367 Favorita, SCS368 Ituporanga, and SC370 Luiza had the shortest roots.

In terms of width, SCS372 Marina and 17025-13 had the widest roots although they were significantly similar (*p* < 0.05) to SCS368 Ituporanga, SCS369 Águas Negras, SCS370 Luiza, and the new varieties Darci, 17007-15, 17052, 17082-8, 17092-9, 17105-20, 17107-18, 17117, 17125-10 and 17162. In addition, the SCS367 Favorita showed the narrowest roots, and its result was statistically similar (*p* < 0.05) to Leandro.

Most of the tuberous roots evaluated were appraised as large, with rounded shapes, elliptical-oblong to elliptical, and relatively thick. In addition, most samples had light brown superficial skin and cream color for the secondary skin. The surfaces of most of the tuberous roots were considered irregular, ranging from smooth to samples with constrictions and protruding veins on their surface. Therefore, sweet potato morphological aspects should be considered for screening the new varieties, since the roots that do not display desirable attributes could be rejected by the consumers during their commercialization. That could lead to the product being discarded and consequently, a decrease in farmer profitability. Individual information about each sample can be observed in Table 2 and Supplementary Figure 1.

In similar studies, Veasey et al. (2007) evaluated the phenotypic and morphological diversity of 74 sweet potato cultivars collected from 30 crops among the São Paulo municipalities of Iguape, Ilha Comprida, and Cananéia, jointly with four commercial cultivars. The authors confirmed that farmers from the evaluated municipalities produced sweet potatoes with a large morphological variation. Therefore, it was recommended that the farmers use healthy sweet potato plants that could be obtained from specialized seed suppliers.

Moulin et al. (2012) used phenotypic descriptors to characterize the morphology of tuberous roots and leaves of 46 sweet potato genotypes produced in Rio de Janeiro. The authors reported that the genetic similarity dendrogram was able to organize the genotypes into 5 distinct groups based on their morphological characteristics. The work emphasized that rural producers in Rio de Janeiro produced sweet potatoes with broad genetic diversity.

The morphological quality, agricultural management, and productivity of new sweet potato plants were essential factors directly related to increasing the supply potential of the crop (Neiva et al., 2011; Oliveira et al., 2015). Thus, the use of selected high-quality plants and good agricultural practices could improve the physical and morphological characteristics of sweet potato plants (Júnior et al., 2009; Rós et al., 2014).

### Sweet Potatoes Pulp Color Profile

Food color is a significant sensory attribute related to intrinsic quality characteristics and product acquisition. Its assessment is also associated with consumer habits surrounding consumption and selection of different food crops (Stich, 2016). Table 3 shows the results obtained from the instrumental color of the pulp fraction of samples.

Based on parameter L* (luminosity), 17092-9 had the highest value which was significantly different (*p* < 0.05) from that of SC370 Luiza and of 17007-15. However, SC370 Luiza and 17007-15 which had the lowest values were significantly similar. It is worth mentioning that cultivar SCS370 Luiza had a dark purple pulp, a characteristic that is directly related to the low value registered for luminosity. Thus, according to color parameter a* samples 17007-15 and SCS370 Luiza had the highest values, as the lowest was recorded for 17162. The 17007-15 exhibited the highest result for parameter b* and it was statistically similar only to SCS367 Favorita which in turn was statistically similar to Darci. Indeed, Darci and SCS370 Luiza parameter b* results were significantly similar (*p* < 0.05).

The highest result for C* was recorded for 17005-15 which was statistically similar to SCS367 Favorita which in turn was similar to SCS371 Katiy, 17092-9, SCS369 Águas Negras, and Darci. The evaluation of h° revealed that purple-fleshed SCS370 Luiza had the highest result which was statistically similar (*p* < 0.05) only to 17092-9 which in turn was similar to 17117. The lowest result observed to h° was for 17007-15 which is similar to 17117. The pulp color of each sweet potato sample can be accessed in Supplementary Figure 1.

Tang et al. (2015) analyzed the bioactive compounds, antioxidant activity, and the instrumental color of five sweet potato varieties with colored pulp (white, yellow, orange, light purple, and dark purple). The roots were subjected to different cooking methods but one of each was kept raw. All samples were lyophilized before the analytical tests. Among the color results reported, the light purple and dark purple samples had great variation. The L* values did not show a great difference between the white, yellow, and orange samples, with small differences between the parameters a* and b*. In comparison, among the 18 samples analyzed, it is possible to subdivide into groups of white color (SCS369 Águas Negras, SCS371 Katiy, Leandro, and 17092-9), cream (Darci, 17082-8, 17125-10, and 17162), yellow (SCS368 Ituporanga and 17052), orange (SCS367 Favorite, SCS372 Marina, 17007-15, 17025-13, 17105-20, 17107-18, and 17117), and purple pulp (SCS370 Luiza). Thus, the group of white pulp was similar for L* close to the value 80, a* show negative values, and b* show similarity close to the value 20. The cream and yellow groups were similar to L*, but with negative a* and higher values of b*. The orange pulp group was slightly lower in L* (results between 57.37 and 79.14), but most were higher in a* and b*.

Pilon et al. (2021) evaluated the bioactive compounds, physical characteristics, and physicochemical composition of eight sweet potato genotypes from Embrapa-Hortaliças (Brazil). With regard to color, samples were grouped into orange, cream, and yellow-fleshed sweet potato, together with two control samples. The orange pulp samples showed results for the L* parameter with values between 71.71 and 76.08, and the cream and yellow group resulted in L* above 80. The orange pulp samples also showed C* values between 48.58 and 51.47, which was higher than the other samples. The results for h° had significant differences between the samples of the same color group, and the cream samples presented higher results than the others (h° 95.03–103.22). These data reported by the authors are in agreement with the values observed for the groups with similar pulp colors (Table 3). On the other hand, the result of h° is smaller than the value observed for the purple pulp sample SCS370 Luiza. Furthermore, the authors communicated that the highest levels of total carotenoids and total phenolic compounds were evaluated for the group of orange-fleshed sweet potatoes.

The occurrence of different colors in sweet potato pulp is linked to the presence of several bioactive compounds that act as pigments, such as anthocyanidins (red/purple), β-carotene (orange), and flavonoids (yellow/orange). In addition to providing color to the food, the bioactive compounds are able to beneficially contribute to the consumer’s metabolism (Mohanraj, 2018; Albuquerque et al., 2019; Amoanimaa-Dede et al., 2020; Alam, 2021).

Notably, color is an attribute of great importance for food products in general. The preference for consumption is related to sensory aspects, and color plays a vital role in the food’s visual appeal. The color of sweet potatoes can be correlated with their physicochemical properties, microbiological quality, and maturation conditions. So, management conditions can directly affect the color aspect in the post-harvest and storage stages. Furthermore, the color impact can be observed in the economic circumstances that involve crop commercialization, as the sweet potatoes that do not have expected quality colors tend to be rejected (Pathare et al., 2013; Prato and Nascimento, 2019; Amoanimaa-Dede et al., 2020).

### Proximal and Total Dietary Fiber Composition

The proximate composition characterization of foods is fundamental to product quality. These characteristics affect consumption, food trade and industrialization, research projects, product development, and decision-making related to collective health actions (Egan et al., 2007). Information regarding the food composition is directly involved in ensuring quality and safety. Therefore, centesimal characterization can benefit consumers concerning food intake options and assist in developing new products (Roe et al., 2017; Mu and Singh, 2019).

The water activity of the samples was all close to the maximum value of 1.00, which is in conformity with the nature of this kind of food (Wojslaw, 2012; Mu and Singh, 2019). The registered cultivars SCS370 Luiza, SCS368 Ituporanga, SCS371 Katiy, SCS372 Marina, and the new varieties 17007-15, 17052, 17092-9, 17117, and 17125-10 had the highest values which were statistically similar to SCS367 Favorita, SCS369 Águas Negras, Leandro, 17025-13, and 17082-8. Sample 17162 had the lowest value for water activity.

Regarding moisture, 17162 had the highest content which was statistically similar to cultivars SCS367 Favorita, SCS368 Ituporanga, SCS369 Águas Negras, and SCS371 Katiy, and for the new varieties Darci, Leandro, 17052, 17105-20, 17117, and 17125-10. The lowest moisture percentages were observed for samples SCS370 Luiza, SCS372 Marina, 17007-15, 17025-13, 17082-8, 17092-9, and 17107-18. Moisture is a critical parameter for the samples’ proximate composition and is directly correlated with the dry matter content of the crop and its shelf life (Damodaran and Parkin, 2017).

The proximal composition data of the sweet potato cultivars and new varieties enabled estimation of the total energy value in kilocalories (Kcal), according to the Atwater conversion values (Nichols, 1994; Welsh, 1994). Sample 17025-13 had the highest variable energy result which was statistically similar (*p* < 0.05) to cultivars SCS368 Ituporanga, SCS370 Luiza, SCS371 Katiy, and SCS372 Marina, and 17007-15, 17082-8, 17092-9, 17107-18, and 17125-10. But the lowest energy result was registered for samples SCS368 Favorita, SCS369 Águas Negras, SCS370 Luiza, SCS371 Katiy, Darci, Leandro, 17052, 17082-8, 17105-20, 17117, 17125-10, and 17162.

Sweet potatoes naturally have a low content of proteins and total lipids in their pulp (Habtemariam, 2019; Mu and Singh, 2019). Among the samples analyzed 17007-15 had the highest protein content that was followed by 17052 which was statistically similar. The lowest protein content was recorded for cultivar SCS372 Marina. Results for total lipids revealed that SCS368 Ituporanga had the highest content which was significantly different (*p* < 0.05) compared to new varieties 17162 and 17107-18. The sample 17007-15 has the highest ash content which result was statistically similar to 17105-20. However, the cultivars SCS371 Katiy, SCS369 Águas Negras, and new varieties Leandro and 17125-10 have the lowest result for this parameter.

The variant SCS372 Marina had the highest content for total carbohydrates followed by 17025-13 and 17107-18. However, the samples SCS369 Águas Negras, Darci, Leandro, 17052, 17007-15, 17117, and 17162 had the lowest content in which its results were statistically similar. For total dietary fiber, results showed that registered cultivar SCS372 Marina was followed by SCS369 Ituporanga, SCS370 Luiza, and SCS371 Katiy, and the new varieties 17007-15, 17025-13, 17092-9, 17082-8, 17105-20, 17107-18, 17117 and 17125-10 which were statistically similar. The lowest results were recorded for SCS368 Favorita, SCS369 Águas Negras, Leandro, Darci, 17052, and 17162.

Dietary fiber plays an important role in the nutritional composition of sweet potatoes. Among its definitions, dietary fiber can be understood as a structural polysaccharide produced by plants that when consumed can resist the endogenous enzymes present in the digestion, which has beneficial effects on human health (Jha et al., 2017; Tiwari and Cummins, 2021). It can be subdivided into soluble and insoluble dietary fiber by means of its solubility. Soluble dietary fiber includes some carbohydrates such as pectin, plant gums, and glucomannan. The insoluble dietary fiber group includes cellulose, hemicellulose, lignin, and chitin (Mu and Singh, 2019).

Rose and Vasanthakaalam (2011) compared the centesimal composition of four sweet potato cultivars in Rwanda, in which two cultivars had yellow pulp and the other two had white pulp. Among all results, moisture content ranged from 62.58 to 64.34%, and the values of total proteins and total reducing sugars were higher for the yellow pulp varieties (the highest values were 0.91 and 2.5, respectively). In addition, the results of crude fiber and ash content were also superior in the yellow pulp samples. The authors informed that the cultivation of yellow pulp sweet potato (especially Kwizekumwe) became interesting since these cultivars were nutritionally superior to the others evaluated. Among the sweet potato registered samples evaluated in the study, SCS370 Luiza and SCS372 Marina have colored pulp, dark purple, and yellowish/orange, respectively.

Alam et al. (2016) estimated the proximal composition, total carotenoids, and total polyphenols in nine orange-fleshed sweet potato cultivars from the Tuber Crops Research Center in Bangladesh. Among the reported results, moisture varied between 70.95 and 72.96%, total protein 1.91–5.83%, total lipid 0.17–0.63%, crude fiber 0.3–0.53%, ash 1.17–1.29%, and total carbohydrates 21.1–24.5%. The authors indicated that the sweet potato cultivars analyzed had a satisfactory content for proteins and carbohydrates, but were low for total lipids. The conclusion suggests that the consumption of orange-fleshed sweet potatoes should be encouraged in Bangladesh as a way of mitigating malnutrition problems.

Santos et al. (2019) evaluated the centesimal and mineral composition of 48 samples of white-fleshed sweet potato, divided between samples from conventional and organic cultivation. The quality of the analytical results was corroborated by statistical tests including principal components analysis and hierarchical cluster analysis. Regarding the proximate composition, the results for the samples of conventional and organic cultivation were for moisture 72–72%, ash 0.87–0.9%, total proteins 1.5–1.4%, total lipids 0.63–0.54%, and total carbohydrates 24.8–23.9%. The authors point out that the results observed with the principal component analysis and hierarchical cluster analysis tests did not show the significant statistical difference, which was suitable for classifying the samples into two distinct groups according to the cultivation method applied for the production of the tuberous roots.

In this context, Arshad et al. (2021) developed a comparative study between Pakistani white-fleshed sweet potato and potato cultivars regarding the proximate composition, mineral content, pulp color, bioactive compounds, as well as antioxidant activity. The samples were lyophilized before the analytical characterization. Among the results published in the study, the white-fleshed sweet potato sample showed maximum moisture contents of 5.57%, ash 1.79%, total lipids 0.86%, total proteins 5.56%, crude fiber 2.06%, and nitrogen-free extract (total carbohydrates) of 83.17%. The authors conclude in their work that the white-fleshed sweet potato had a higher proximate composition, mineral and antioxidant activity when compared to the white-fleshed potato.

In their review work, Amagloh et al. (2021) highlight that the increase in the consumption of foods containing dietary fiber is related to the decrease in the prevalence of chronic non-communicable diseases by stimulating beneficial effects on the consumer’s health. The authors report that sweet potato is a food rich in fiber in which the content of this nutrient can reach 3 g in 100 g of fresh weight. Regardless of the variety, sweet potato is reported to be beneficial for patients with type 2 diabetes mellitus due to its dietary fiber content and moderate glycemic index. This information corroborates the results for the total dietary fiber analysis obtained in the study. Based on the cultivar SCS372 Marina, the total fiber content on a dry basis was 12.6 g ± 1.26, which on a fresh basis is around 2.90 g in 100 g of pulp.

These data were in agreement with the results found in the present study. It is noteworthy that the analyzes of water activity and moisture were performed on the fresh portion of the 18 sweet potato samples, and these values ranged from 0.97–0.99 and 76.28–83.32%, respectively. The analyzes of total proteins, total lipids, ash, total dietary fibers, and total lipids were performed with the lyophilized pulp. In general, the values observed for the proximal composition of the sweet potato cultivars and new varieties used in this study were similar, or even higher to other results reported in the literature (Júnior et al., 2005, 2012; Universidade Estadual De Campinas [UNICAMP], 2011; Melo et al., 2011; Universidade Federal De São Paulo [USP], 2020).

### Screening of Sweet Potato Cultivars and New Varieties

The realization of the first FA was necessary to verify which input independent variables did not hold an adequate communality value, so they could be removed from the input data. For this reason, it was necessary to carry out a second FA to corroborate the independent variables with greater communality. As a result, the second exploratory FA was able to compute 92.08% of the explanatory variability of the input data, which corroborate analysis best fit. Thus, Factor 1 explains 27.96% of the data variability, while Factor 2 explains 23.04%, Factor 3 18.61%, Factor 4 14.42%, and Factor 5 8.06%. Supplementary Table 7 shows the relationship between the variables and factors obtained in the second exploratory FA, and Supplementary Figure 3 illustrates the eigenvalues of the variables used in the second FA.

According to Supplementary Table 7, the second exploratory FA reveals that M, Ev, Carb, and Tdf are related to Factor 1 and correlated to each other. The parameters L* and a* are related to Factor 2. Variables b*, C*, and h° are related to Factor 3, and Wa and Ash are correlated to Factor 4. Finally, the morphology variables We and Le have a high relationship with Factor 5.

Factor 1 has the highest explanatory rate of the secondary FA, which is described as an index related to the nutritional quality of sweet potato cultivars and new varieties. The independent variables aggregated to Factor 1 hold great importance for the consumers which desire nutritious foods. Factors 2, 3, and 4 can be interpreted as a comparison index between the instrumental color parameters and the proximal composition attributes. However, Factor 5 has great relevance for the study, which aggregates We and Le morphology variables. These variables are essential for agricultural producers who intend to grow sweet potatoes with economic goals. The financial value of the sweet potato crop is based on the total weight in tons of its production. The coefficient clustering graph for the 13 variables of the second exploratory FA as a function of the 1 × 2 × 5 Factors is presented in Figure 1.

The Supplementary Figure 2 confirms that the five Factors generated in the second exploratory FA hold an eigenvalue above 1 (Kaiser, 1958). Furthermore, according to the Scree-plot Eigenvalue Diagram Criterion (Cattell, 1966; Woods and Edwards, 2011), the number of factors to be kept in the factor analysis must stand before the inflection point of the factor curve, which is in accordance with Supplementary Figure 3. The elbow is observed after Factor 5, which corroborates the agreement of the analysis.

The Supplementary Table 8 illustrates the communality values of the 5 Factors generated in the second FA with the 13 previously established input variables. Supplementary Table 8 also confirms the adequacy of exploratory FA according to independent variables of more significant communality along with the calculated factors. The communality of the 13 independent variables used in the second FA has values >0.7 in the column of the fifth factor.

It can be observed that We, Le, Wa, M, Ev, Carb, Tdf, Ash, L*, a*, b*, C*, and h° are associated with the total explained variance relative to the samples dependent variable. It is precisely these 13 variables that were submitted as input data for the PCA. Thus, the second exploratory FA explained 92.08% of the variability embedded in the input data. The non-explanatory percentage of the model is 7.92%, which is related to the specific variance of the input data incorporated in the random error of the model.

Regarding the results of the PCA test, it is possible to notice that the We and Le variables have an approximate communality score of 0.7 (0.66 ≈ 0.7) in the column of PC 4 (Supplementary Table 13). It is noteworthy that these two independent variables related to the morphology of the samples derive as a direct demand from rural producers who plant and sell sweet potatoes. Agronomic traits are some elements that influence the farmer’s decision to choose the sweet potato varieties to be planted (Kassali, 2011; Prakash et al., 2018). The sweet potato morphological quality is also related to product acceptance by consumers. Therefore, these independent variables are related to crop economic value. This circumstance corroborates the presence of the We and Le independent variables at this stage of exploratory statistical analysis. Figure 2 illustrates the dispersion of independent variables as a function of the principal components calculated by the PCA.

It is noteworthy that in Figure 3, graph (A) has 72.22% of the samples fully immersed within some quadrant. Graph (B) has 66.66% of the samples within some quadrant, whereas graph (C) has 55.55%. Among the information that derives from Figures 2, 3, it is essential to track the positions of the independent variables of most significant interest and make the cross-reference with the sample distribution.

The independent variables with the greatest significance are clustered by Carb, Tdf, and Ev as the nutritional quality index and We and Le as the morphology index. A careful evaluation reveals that the SCS372 Marina and 17025-13 samples own a dispersion relationship with the variables related to nutritional quality. At some point in their dispersion, the samples SCS370 Luiza, SCS372 Marina, 17125-10, and 17117 have a relationship with the independent variables that make up the morphological attributes of the samples.

## Conclusion

The 18 sweet potato samples were analyzed for their morphology, pulp color, proximal composition, and total dietary fiber content. Out of the 16 independent variables, the exploratory factor analysis identified 92.08% of assertiveness the 13 independent variables with communality >0.7. The PCA generated 4 PC to clarify 84.01% of the data’s explanatory variance. Among the 6 cultivars registered, the SCS372 Marina and SCS370 Luiza have the aptitude to be recommended to farmers for crop production. From the 12 sweet potato new varieties, 17025-13, 17125-10, and 17117 showed high potential to be patent and registration, for in the future be available to farmers. Moreover, analytical results corroborate the nutritional quality of sweet potato cultivars and new varieties applied in the study.

## Data Availability Statement

The datasets presented in this study can be found in online repositories. The names of the repository/repositories and accession number(s) can be found in the article/Supplementary Material.

## Author Contributions

CL: samples harvesting, analytical procedures, statistical analysis, and article writing, review, and publication. BS: analytical procedures. AF: article writing and review. CM, GW, and DA: samples breeding, field trials, planting, and harvesting. All authors listed have made a substantial, direct, and intellectual contribution to the work and approved it for publication.

## Conflict of Interest

The authors declare that the research was conducted in the absence of any commercial or financial relationships that could be construed as a potential conflict of interest.

## Publisher’s Note

All claims expressed in this article are solely those of the authors and do not necessarily represent those of their affiliated organizations, or those of the publisher, the editors and the reviewers. Any product that may be evaluated in this article, or claim that may be made by its manufacturer, is not guaranteed or endorsed by the publisher.

## References

[B1] Abdissa T. Dechassa N. Alemayehu Y. (2012). Sweet potato growth parameters as affected by farmyard manure and phosphorous application at Adami Tulu, Central Rift Valley of Ethiopia. *Agric. Sci. Res. J.* 2 1–12. 10.11648/j.plant.20190701.11

[B2] Alam M. K. (2021). A comprehensive review of sweet potato (*Ipomoea batatas* [L.] Lam): revisiting the associated health benefits. *Trends Food Sci. Technol.* 115 512–529. 10.1016/j.tifs.2021.07.001

[B3] Alam M. K. Rana Z. H. Islam S. N. (2016). Comparison of the proximate composition, total carotenoids and total polyphenol content of nine orange-fleshed sweet potato varieties grown in Bangladesh. *Foods* 5 64. 10.3390/foods5030064 28231159 PMC5302402

[B4] Albuquerque T. M. R. Sampaio K. B. Souza E. L. (2019). Sweet potato roots: unrevealing an old food as a source of health promoting bioactive compounds–a review. *Trends Food Sci. Technol.* 85 277–286. 10.1016/j.tifs.2018.11.006

[B5] Alkarkhi A. F. M. Alqaraghuli W. A. A. (2019). “Chapter 9: Factor Analysis,” in *Easy Statistics for Food Science with R*, eds Alkarkhi A. F. M. Alqaraghuli W. A. A. (London: Academic Press), 143–159. 10.1016/B978-0-12-262-2.00009-1

[B6] Amagloh F. C. Yada B. Tumuhimbise G. A. Amagloh F. K. Kaaya A. N. (2021). The potential of sweetpotato as a functional food in sub-saharan africa and its implications for health: a review. *Molecules* 26 2971. 10.3390/molecules26102971 34067782 PMC8156662

[B7] American Association of Cereal Chemists [AACC] (2010). *International, “Approved Methods of Analysis”*, 11th Edn. St. Paul, MN: AACC International.

[B8] Amoanimaa-Dede H. Su C. Akwasi Y. Chen C. Yang S. Zhu H. (2020). Flesh color diversity of sweet potato: an overview of the composition, functions, biosynthesis, and gene regulation of the major pigments. *Phyton (Buenos Aires)* 89 805–833. 10.32604/phyton.2020.011979

[B9] Armanino C. Forina M. Gardiner P. H. E. Heuvel E. J. Kateman G. Lanteri S. (1987). *Chemometrics and Species Identification.* New York, NY: Springer-Verlag, 181.

[B10] Arshad A. Iqbal H. Siddiqa A. Zulfiqar T. Tareen M. B. Amna D. (2021). Comparative study of potato (*Solanum tuberosum* L.) and sweet potato (*Ipomoea batatas* L.): evaluation of proximate composition, polyphenol content, mineral and antioxidant activities. *Appl. Sci.* 11 11844. 10.3390/app112411844

[B11] Association of Agricultural Chemists [AOAC] (2005). *Official Method of Analysis of the Association of Analytical Chemists*, 18th Edn. Washington DC: AOAC.

[B12] Caivano J. L. Buera M. P. (2016). *Color in Food Technological and Psychophysical Aspects.* Boca Raton, FL: CRC Press, 478.

[B13] Cartabiano C. E. L. Porcu O. M. De Casas A. F. (2020). Sweet potato (*Ipomoea batatas* L. Lam) nutritional potential and social relevance: a review. *Int. J. Eng. Res. Appl.* 10 23–40. 10.9790/9622-1006082340

[B14] Cattell R. B. (1966). The scree-test for the number of factors. *Multivariate Behav. Res.* 1 245–276. 10.1207/s15327906mbr0102_10 26828106

[B15] Chen H. Sun J. Liu J. Gou Y. Zhang X. Wu X. (2019). Structural characterization and anti-inflammatory activity of alkali-soluble polysaccharides from purple sweet potato. *Int. J. Biol. Macromol.* 131 484–494. 10.1016/j.ijbiomac.2019.03.126 30904524

[B16] Coordenadoria De Desenvolvimento Dos Agronegócios [CODEAGRO] (2014). *Batata-doce: Normas de Classificação. Programa Brasileiro para Modernização da Horticultura*, Vol. 12. Barreiras: CODEAGRO.

[B17] Cruz I. C. Topa M. A. (2009). *Multivariate Analysis as a Supplier Management Tool Aiming at a Relationship with Competitive Advantage*. Monograph (Bachelor of Statistics). Curitiba: Federal University of Paraná.

[B18] Damodaran S. Parkin K. L. (2017). *Química de Alimentos de Fennema-5ed.* Porto Alegre: Artmed Editora, 1120.

[B19] De Jonge B. Salazar R. Visser B. (2021). How regulatory issues surrounding new breeding technologies can impact smallholder farmer breeding: a case study from the Philippines. *Plants People Planet* 4 96–105. 10.1002/ppp3.10219

[B20] Dutta S. (2015). Sweet potatoes for diabetes mellitus: a systematic review. *Pharmacophore* 6 60–72.

[B21] Egan M. B. Fragodt A. Raats M. M. Hodgkins C. Lumbers M. (2007). The importance of harmonizing food composition data across Europe. *Eur. J. Clin. Nutr.* 61 813–821. 10.1038/sj.ejcn.1602823 17554245

[B22] Federizzi L. C. Carbonell S. A. M. Pacheco M. T. Nava I. C. (2012). Breeders’ work after cultivar development: the stage of recommendation. *Crop Breed. Appl. Biotechnol.* 12 67–74. 10.1590/S1984-70332012000500008

[B23] Food and Agriculture Organization of the United Nations (2021). *FAOSTAT Statistical Database.* Available online at: https://www.fao.org/faostat/en/ (accessed November 25, 2021)

[B24] Forina M. Leardi R. Armanino C. Lanteri S. (2014). *PARVUS – An Extendible Package for Data Exploration, Classification and Correlation.* Genoa: Institute of Pharmaceutical and Food Analysis and Technologies. 10.1002/cem.1180040210

[B25] Gasura E. Matsaure F. Setimela P. S. Rugare J. T. Nyakurwa C. S. Andrade M. (2021). Performance, variance components, and acceptability of pro-vitamin A-biofortified sweetpotato in Southern Africa and implications in future breeding. *Front. Plant Sci.* 12:696738. 10.3389/fpls.2021.696738 34539691 PMC8446612

[B26] Grüneberg W. J. Ma D. Mwanga R. O. M. Carey E. E. Huamani K. Diaz F. (2015). “Advances in sweetpotato breeding from 1992 to 2012,” in *Potato and Sweetpotato in Africa. Transforming the Value Chains for Food and Nutrition Security*, eds Low J. Nyongesa M. Quinn S. Parker M. (Oxfordshire: CABI International), 3–68. 10.1079/9781780644202.0003

[B27] Habtemariam S. (2019). “Other common and exotic foods with growing importance as antidiabetic agents,” in *Medicinal Foods as Potential Therapies for Type-2 Diabetes and Associated Diseases: The Chemical and Pharmacological Basis of their Action*, ed. Habtemariam S. (London: Academic Press), 985–1047. 10.1016/b978-0-08-102922-0.00025-0

[B28] Heo S. Choi J. Y. Kim J. Moon K. D. (2021). Prediction of moisture content in steamed and dried purple sweet potato using hyperspectral imaging analysis. *Food Sci. Biotechnol.* 30 783–791. 10.1007/s10068-021-00921-z 34249383 PMC8225792

[B29] Huaman Z. (1991). *Descriptors for Sweet Potato. International Board for Plant Genetic Resources-IBPGR, Centro Internacional de la Papa-CIP.* Tainan: Asian Vegetable Research and Development Center-AVRDC, 134.

[B30] Jha S. K. Singh H. R. Prakash P. (2017). “Dietary fiber and human health: an introduction,” in *Dietary Fiber for the Prevention of Cardiovascular Disease Fiber’s Interaction Between Gut Micoflora, Sugar Metabolism, Weight Control and Cardiovascular Health*, ed. Samaan R. A. (London: Academic Press), 1–22. 10.1016/b978-0-12-805130-6.00001-x

[B31] Ju J. H. Yoon H. S. Park H. J. Kim M. Y. Shin H. K. Park K. Y. (2011). Anti-obesity and antioxidative effects of purple sweet potato extract in 3T3-L1 adipocytes in vitro. *J. Med. Food* 14 1097–1106. 10.1089/jmf.2010.1450 21861722

[B32] Júnior A. J. L. S. Praça E. F. Grangeiro L. C. Braga A. P. Menezes M. A. Silva A. R. (2005). “Composição centesimal de cultivares de batata-doce colhidas aos quatro meses,” in *Proceedings of the Anais do 45*^a^* Congresso Brasileiro de Oleicultura – CBO.*

[B33] Júnior C. A. Viana D. J. S. Pinto N. A. Ribeiro K. G. Pereira R. C. Neiva I. P. (2012). Características produtivas e qualitativas de ramas e raízes de batata-doce. *Hortic. Bras.* 30 584–589. 10.1590/S0102-05362012000400004

[B34] Júnior V. C. A. Viana D. J. S. Fernandes J. S. C. Figueiredo J. A. Nunes U. R. Neiva I. P. (2009). Selection of sweet potato clones for the region of Alto Vale do Jequitinhonha. *Hortic. Bras.* 27 389–393. 10.1590/S0102-05362009000300024

[B35] Kaiser H. F. (1958). The varimax criterion for analytic rotation in factor analysis. *Psychometrika* 23 187–200. 10.1007/BF02289233

[B36] Kaiser H. F. (1970). A second generation little jiffy. *Psychometrika* 35 401–415. 10.1007/BF02291817

[B37] Karuri H. W. Ateka E. M. Amata R. Nyende A. B. Muigai A. W. T. Mwasame E. (2010). Evaluating diversity among Kenyan sweet potato genotypes using morphological and SSR markers. *Int. J. Agric. Biol.* 12 33–38.

[B38] Kassali R. (2011). Economics of sweet potato production. *Int. J. Veg. Sci.* 17 313–321. 10.1080/19315260.2011.553212

[B39] Katayama K. Kobayashi A. Sakai T. Kuranouchi T. Kai Y. (2017). Recent progress in sweetpotato breeding and cultivars for diverse applications in Japan. *Breed. Sci.* 97 3–14. 10.1270/jsbbs.16129 28465663 PMC5407919

[B40] Kim H. J. Koo K. A. Park W. S. Kang D. M. Kim H. S. Lee B. Y. (2020). Anti-obesity activity of anthocyanin and carotenoid extracts from color-fleshed sweet potatoes. *J. Food Biochem.* 44 e13438. 10.1111/jfbc.13438 32812262

[B41] Konica Minolta Corporation (1994). *Precise Color Communication: Color Control From Feeling to Instrumentation.* Chiyoda: Konica Minolta Sensing Americas, Inc, 62.

[B42] Konica Minolta Corporation (2017). *Understanding the CIE L*C*h Color Space.* Chiyoda: Konica Minolta Sensing Americas, Inc.

[B43] Labrada H. R. (2009). “Participatory seed diffusion: experiences from the field,” in *Plant breeding and farmer participation*, eds Ceccarelli S. Guimarães E. P. Weltzein E. (Rome: Food and Agriculture Organization of the United Nations), 589–612.

[B44] Li P. G. Mu T. H. Deng L. (2013). Anticancer effects of sweet potato protein on human colorectal cancer cells. *World J. Gastroenterol.* 19 3300–3308. 10.3748/wjg.v19.i21.3300 23745032 PMC3671082

[B45] Low J. W. Arimond M. Osman N. Cunguara B. Zano F. Tschirley D. (2007). A food-based approach introducing orange-fleshed sweet potatoes increased vitamin A intake and serum retinol concentrations in young children in rural Mozambique. *J. Nutr.* 137 1320–1327. 10.1093/jn/137.5.1320 17449599

[B46] Low J. W. Ortiz R. Vandamme E. Andrade M. Biazin B. Grüneberg W. J. (2020). Nutrient-dense orange-fleshed sweetpotato: advances in drought-tolerance breeding and understanding of management practices for sustainable next-generation cropping systems in sub-Saharan Africa. *Front. Sustain. Food Syst.* 4:50. 10.3389/fsufs.2020.00050

[B47] Meira M. Silva E. P. D. David J. M. David J. P. (2012). Review of the genus Ipomoea: traditional uses, chemistry, and biological activities. *Rev. Bras. Farmacogn.* 22 682–713. 10.1590/S0102-695X2012005000025

[B48] Melo W. F. Gomes L. M. Moita A. W. Amaro G. B. Bessa F. P. Dusi A. N. (2011). “Biofortificação no Brasil (BioFort: avaliação preliminar de clones de batata-doce ricos em betacaroteno,” in *Proceedings of the Anais do 51^°^ Congresso Brasileiro de Olericultura*, Viçosa, 2675–2680.

[B49] Mohanraj R. (2018). “sweet potato: bioactive compounds and health benefits,” in *Bioactive Molecules in Food. Reference Series in Phytochemistry*, eds Mérillon J. M. Ramawat K. (Cham: Springer), 1–16. 10.1007/978-3-319-54528-8_62-1

[B50] Mohanraj R. Sivasankar S. (2014). Sweet potato (*Ipomoea batatas* [L.] Lam)-A valuable medicinal food: a review. *J. Med. Food* 17 733–741. 10.1089/jmf.2013.2818 24921903

[B51] Moulin M. M. Rodrigues R. Gonçalves L. S. A. Sudré C. P. Santos M. H. Silva J. R. P. (2012). Collection and morphological characterization of sweet potato landraces in north of Rio de Janeiro state. *Hortic. Bras.* 30 286–292. 10.1590/S0102-05362012000200017

[B52] Mu T. Singh J. (Eds). (2019). *Sweet Potato: Chemistry, Processing, and Nutrition.* London: Academic Press, 400. 10.1016/C2016-0-05204-X

[B53] Mu T. Sun H. Zhang M. Wang C. (2017). *Sweet Potato Processing Technology.* London: Academic Press, 429.

[B54] Mugumaarhahama Y. Mondo J. M. Cokola M. C. Ndjadi S. S. Mutwedu V. B. Kazamwali L. M. (2021). Socio-economic drivers of improved sweet potato varieties adoption among smallholder farmers in South-Kivu Province, DR Congo. *Sci. Afr.* 12:e00818. 10.1016/j.sciaf.2021.e00818

[B55] Mwanga R. O. M. Swanckaert J. Pereira G. S. Andrade M. I. Makunde G. Grüneberg W. J. (2021). Breeding progress for vitamin A, Iron and Zinc biofortification, drought tolerance, and sweetpotato virus disease resistance in sweetpotato. *Front. Sustain. Food Syst.* 5:616674. 10.3389/fsufs.2021.616674

[B56] Neiva I. P. de Andrade Júnior V. C. Viana D. J. S. Figueiredo J. A. Mendonça Filho C. V. Parrella R. A. (2011). Caracterização morfológica de acessos de batata-doce do banco de germoplasma da UFVJM, Diamantina. *Hortic. Bras.* 29 537–541. 10.1590/S0102-05362011000400016

[B57] Nichols B. L. (1994). Atwater and USDA nutrition research and service: a prologue of the past century. *J. Nutr.* 124 1718S–1727S. 10.1093/jn/124.suppl_9.1718S 8089739

[B58] Oliveira C. D. Souza A. F. Duda P. R. O. Souza A. A. D. F. (2015). Produtividade de cultivares de batata-doce, plantadas com ramas de safra anterior, conservadas durante o inverno em diferentes ambientes. *Rev. Técnico Cient. IFSC* 5 234–241.

[B59] Pathare P. B. Opara U. L. Al-Said F. A. (2013). Colour measurement and analysis in fresh and processed foods: a review. *Food Bioprocess Technol.* 6 36–60. 10.1007/s11947-012-0867-9

[B60] Pilon L. Guedes J. S. Bitencourt B. S. Melo R. A. D. C. Vendrame L. P. Amaro G. B. (2021). Quality characterization, phenolic and carotenoid content of new orange, cream and yellow-fleshed sweetpotato genotypes. *Hortic. Bras.* 39 299–304. 10.1590/s0102-0536-20210309

[B61] Prakash P. Kishore P. Jaganathan D. Immanual S. Sivakumar P. S. (2018). “The status, performance and impact of sweet potato cultivation on farming communities of Odisha, India,” in *Proceedings of the 2018 International Association of Agricultural Economists*, Vancouver, BC. 10.22004/ag.econ.277216

[B62] Prato T. S. Nascimento M. G. (2019). Influência da cor e do odor na discriminação do sabor de um produto. *Inovação Ciênc. Tecnol. Alimentos.* 179–186. 10.22533/at.ed.00019091019

[B63] Qaim M. (2020). Role of new plant breeding technologies for food security and sustainable agricultural development. *Appl. Econ. Perspect. Policy* 42 129–150. 10.1002/aepp.13044

[B64] Ricachenevsky F. K. Vasconcelos M. W. Shou H. Johnson A. A. T. Sperotto R. A. (2019). Improving the nutritional content and quality of crops: promises, achievements, and future challenges. *Front. Plant Sci.* 10:738. 10.3389/fpls.2019.00738 31244870 PMC6563719

[B65] Rós A. B. Narita N. Horata A. C. S. (2014). Produtividade de batata-doce e propriedades físicas e químicas de solo em função de adubação orgânica e mineral. *Ciênc. Agrár. Londrina* 35 205–214. 10.5433/1679-0359.2014v35n1p205

[B66] Roe M. Plumb J. Charrondierre U. R. Finglas P. (2017). “Chapter 5: food composition,” in *Public Health Nutrition*, 2nd ed., (John Wiley & Sons Ltd., The Nutrition Society), 36–46.

[B67] Rose I. M. Vasanthakaalam H. (2011). Comparison of the nutrient composition of four sweet potato varieties cultivated in Rwanda. *Am. J. Food Nutr.* 1 34–38. 10.5251/ajfn.2011.1.1.34.38

[B68] Rstudio Team (2019). *RStudio: Integrated Development for R.* Boston, MA: RStudio, Inc.

[B69] Ruttarattanamongkol K. Chittrakorn S. Weerawatanakorn M. Dangpium N. (2016). Effect of drying conditions on properties, pigments and antioxidant activity retentions of pretreated orange and purple-fleshed sweet potato flours. *J. Food Sci. Technol.* 53 1811–1822. 10.1007/s13197-015-2086-7 27413208 PMC4926894

[B70] Santos A. M. D. Lima J. S. Dos Santos I. F. Silva E. F. de Santana F. A. de Araujo D. G. (2019). Mineral and centesimal composition evaluation of conventional and organic cultivars sweet potato (*Ipomoea batatas* (L.) Lam) using chemometric tools. *Food Chem.* 273 166–171. 10.1016/j.foodchem.2017.12.063 30292364

[B71] Singh D. P. Singh A. K. Singh A. (eds). (2021). “Participatory plant breeding,” in *Plant Breeding and Cultivar Development* (London: Academic Press), 483–495. 10.1016/B978-0-12-817563-7.00013-1

[B72] Singh G. (2019). *Plant Systematics: An Integrated Approach.* Boca Raton, FL: CRC Press, 568.

[B73] Srinivas T. Nedunchezhiyan M. (2020). The nexus between adoption and diffusion of production technologies with yield: Evidence from sweet potato farmers in India. *Technol. Soc.* 60 101208. 10.1016/j.techsoc.2019.101208

[B74] Statsoft Inc (2021). *TIBCO Statistica§Ultimate Academic. Version 14.0.0.*

[B75] Stich E. (2016). “Food color and coloring food: quality, differentiation and regulatory requirements in the European Union and the United States,” in *Handbook on Natural Pigments in Food and Beverages Industrial Applications for Improving Food Color*, eds Carle R. Schweiggert R. M. (Duxford: Woodhead Publishing), 3–27.

[B76] Sugata M. Lin C. Y. Shih Y. C. (2015). Anti-inflammatory and anticancer activities of Taiwanese purple-fleshed sweet potatoes (*Ipomoea batatas* L. Lam) extracts. *Biomed Res. Int.* 2015 768093. 10.1155/2015/768093 26509161 PMC4609785

[B77] Sun Y. Liu Y. Yu H. Xie A. Li X. Yin Y. (2017). Non-destructive prediction of moisture content and freezable water content of purple-fleshed sweet potato slices during drying process using hyperspectral imaging technique. *Food Anal. Methods* 10 1535–1546. 10.1007/s12161-016-0722-0

[B78] Tanaka M. Ishiguro K. Oki T. Okuno S. (2017). Functional components in sweetpotato and their genetic improvement. *Breed. Sci.* 61 52–61. 10.1270/jsbbs.16125 28465668 PMC5407917

[B79] Tang Y. Cai W. Xu B. (2015). Profiles of phenolics, carotenoids and antioxidative capacities of thermal processed white, yellow, orange and purple sweet potatoes grown in Guilin, China. *Food Sci. Hum. Wellness* 4 123–132. 10.1016/j.fshw.2015.07.003

[B80] Tiwari U. Cummins E. (2021). “Legume fiber characterization, functionality, and process effects,” in *Pulse Foods: Processing, Quality and Nutraceutical Applications*, eds Tiwari B. Gowen A. McKenna B. (London: Academic Press), 147–175. 10.1016/B978-0-12-818184-3.00007-6

[B81] Truong V. D. Avula R. Y. Pecota K. V. Yencho G. C. (2018). “Sweetpotato production, processing, and nutritional quality,” in *Handbook of vegetables and vegetable processing*, eds Siddiq M. Uebersax M. A. (Hoboken, NJ: John Wiley & Sons Ltd), 811–838. 10.1002/9781119098935.ch35

[B82] Universidade Estadual De Campinas [UNICAMP] (2011). *Tabela Brasileira de Composição de Alimentos-TACO.* Brazil: Núcleo de Estudos e Pesquisas em Alimentação–NEPA, 161.

[B83] Universidade Federal De São Paulo [USP] (2020). *Tabela de Composição Química dos Alimentos (TABNUT). Escola Paulista de Medicina.* São Paulo: Departamento de Informática em Saúde, Universidade Federal de São Paulo.

[B84] Van Jaarsveld P. J. Faber M. Tanumihardjo S. A. Nestel P. Lombard C. J. Benadé A. J. S. (2005). β-Carotene–rich orange-fleshed sweet potato improves the vitamin A status of primary school children assessed with the modified-relative-dose-response test. *Am. J. Clin. Nutr.* 81 1080–1087. 10.1093/ajcn/81.5.1080 15883432

[B85] Veasey E. A. Silva J. R. Q. Rosa M. S. Borges A. Bressan E. A. Peroni N. (2007). Phenology and morphological diversity of sweet potato (*Ipomoea batatas* landraces of the Vale do Ribeira. *Sci. Agric.* 64 416–427. 10.1590/S0103-90162007000400013

[B86] Wadl P. A. Olukolu B. A. Branham S. E. Jarret R. L. Yencho G. C. Jackson D. M. (2018). Genetic diversity and population structure of the USDA sweetpotato (*Ipomoea batatas*) germplasm collections using GBSpoly. *Front. Plant Sci.* 9:1166. 10.3389/fpls.2018.01166 30186293 PMC6111789

[B87] Wang S. Nie S. Zhu F. (2016). Chemical constituents and health effects of sweet potato. *Food Res. Int.* 89 90–116. 10.1016/j.foodres.2016.08.032 28460992

[B88] Welsh S. (1994). Atwater to the present: evolution of nutrition education. *J. Nutr.* 124 1799S–1807S. 10.1093/jn/124.suppl_9.1799S 8089752

[B89] Weltzien E. Rattunde F. Christinck A. Isaacs K. Ashby J. (2019). Gender and farmer preferences for varietal traits: evidence and issues for crop improvement. *Plant Breed. Rev.* 43 243–278.

[B90] Wojslaw E. B. (2012). *Food Technology.* Federal District, Brasilia, Brazil. Available online at: https://docplayer.com.br/4139684-Tecnologia-de-alimentos.html

[B91] Woods C. M. Edwards M. C. (2011). “Factor analysis and related methods,” in *Essential Statistical Methods for Medical Statistics*, eds Rao C. R. Miller J. P. Rao D. C. (Burlington, MA: Elsevier), 174–201. 10.1016/b978-0-444-53737-9.50009-8

[B92] Yuan B. Yang X. Q. Kou M. Lu C. Y. Wang Y. Y. Peng J. (2017). Selenylation of polysaccharide from the sweet potato and evaluation of antioxidant, antitumor, and antidiabetic activities. *J. Agric. Food Chem.* 65 605–617. 10.1021/acs.jafc.6b04788 28052202

