# Supplementary material for: Sweet Potato New Varieties Screening Based on Morphology, Pulp Color, Proximal Composition, and Total Dietary Fiber Content via Factor Analysis and Principal Component Analysis
Source: Front Plant Sci. 2022 May 5;13:852709. doi: 10.3389/fpls.2022.852709 (PMC9119308; doi:10.3389/fpls.2022.852709)
Supplement: Supplementary file 1 [file Data_Sheet_1.docx]

Supplementary Material

| 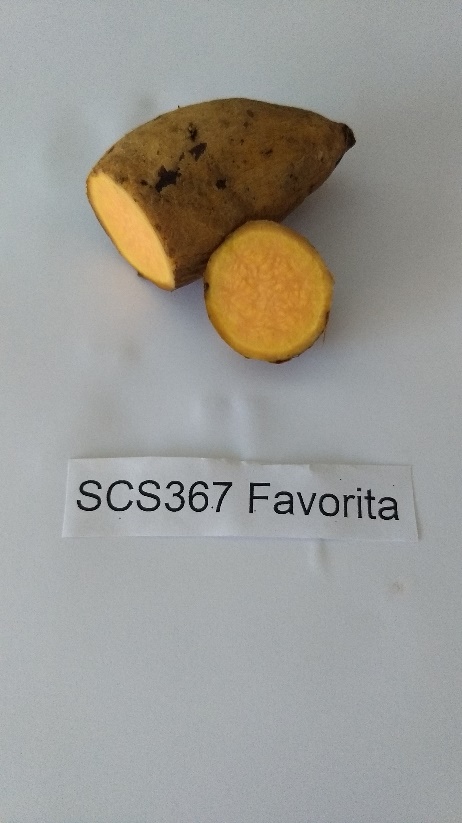 | 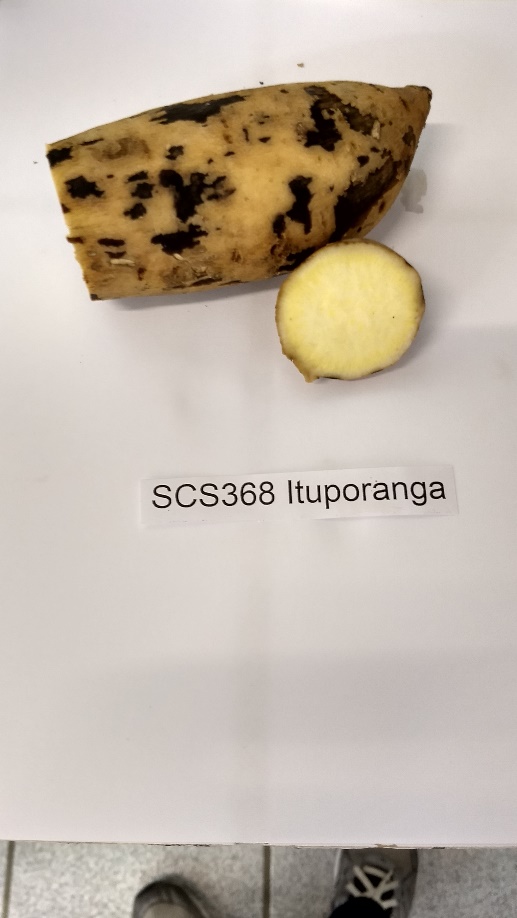 | 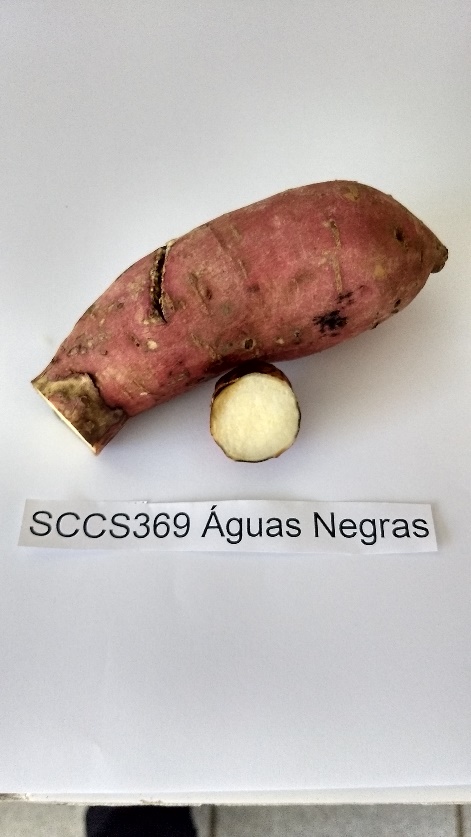 |
| --- | --- | --- |
| 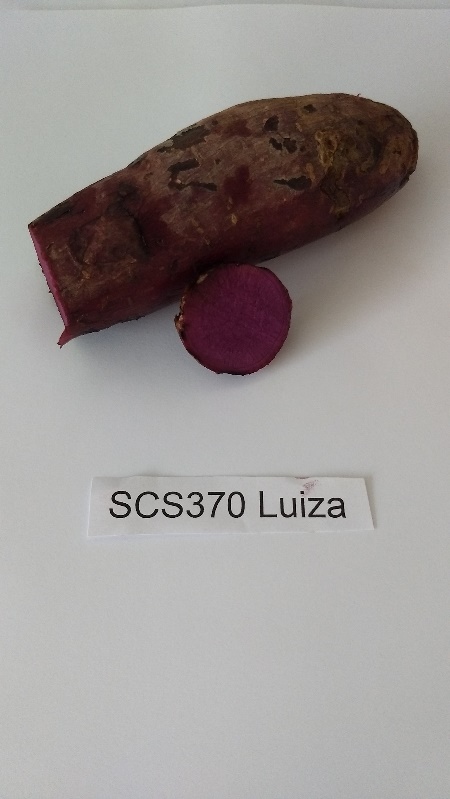 | 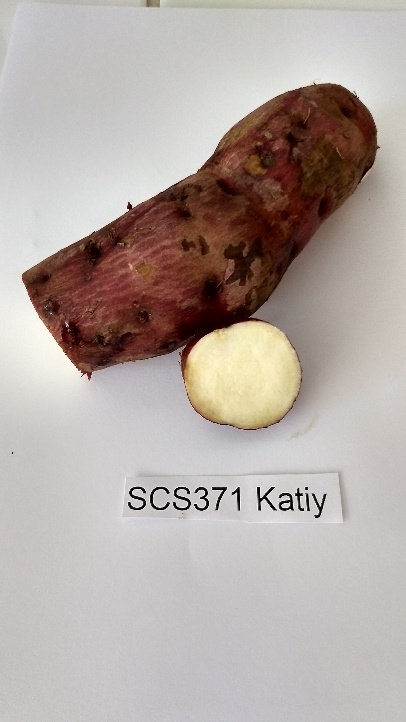 | 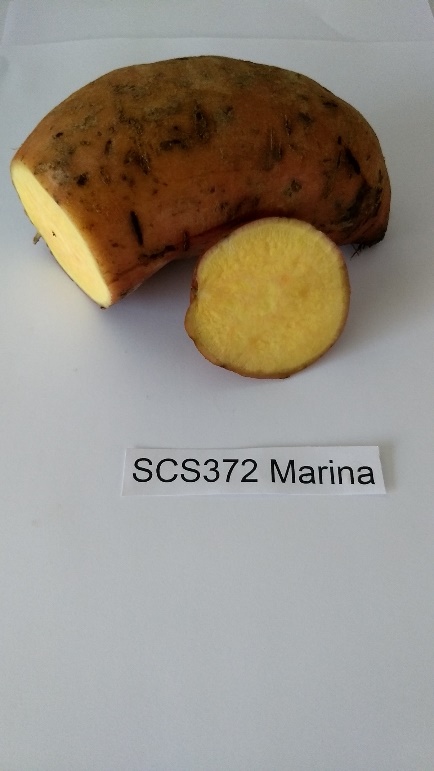 |
| 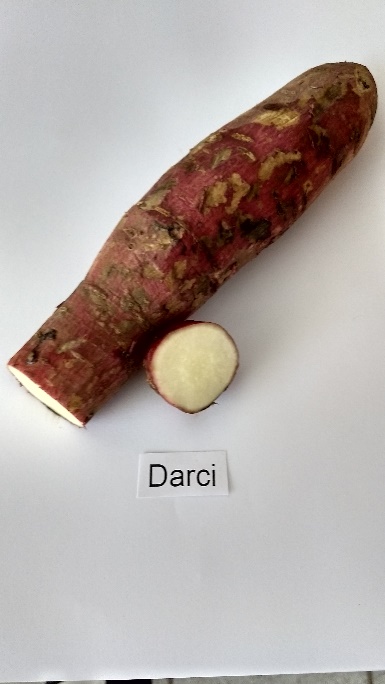 | 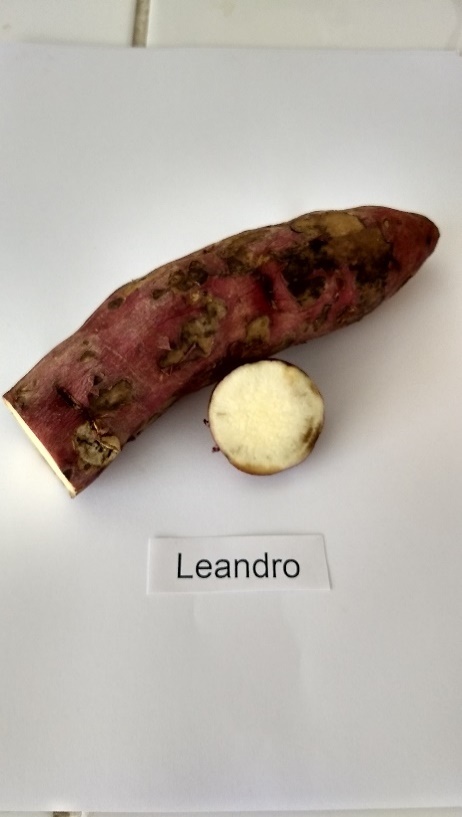 | 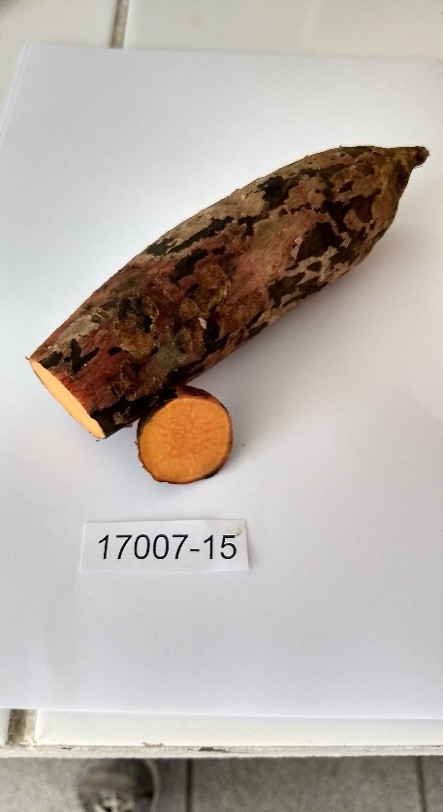 |
| 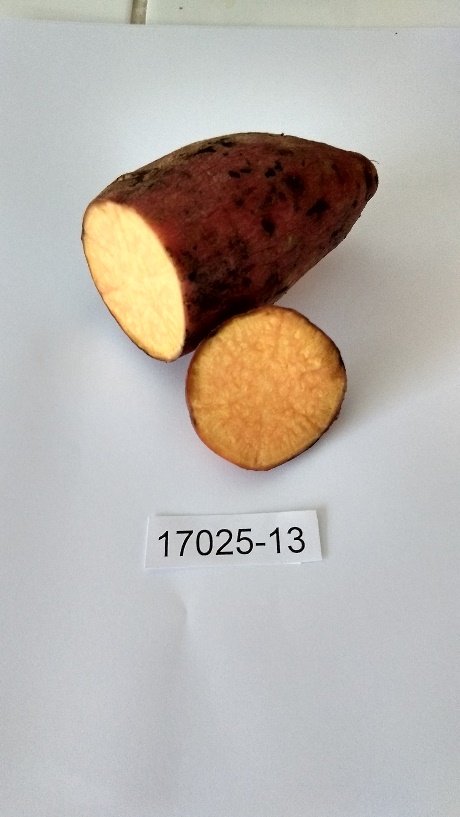 | 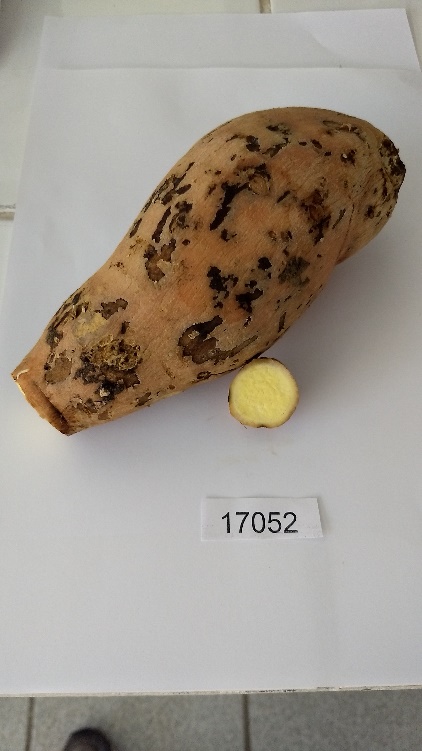 | 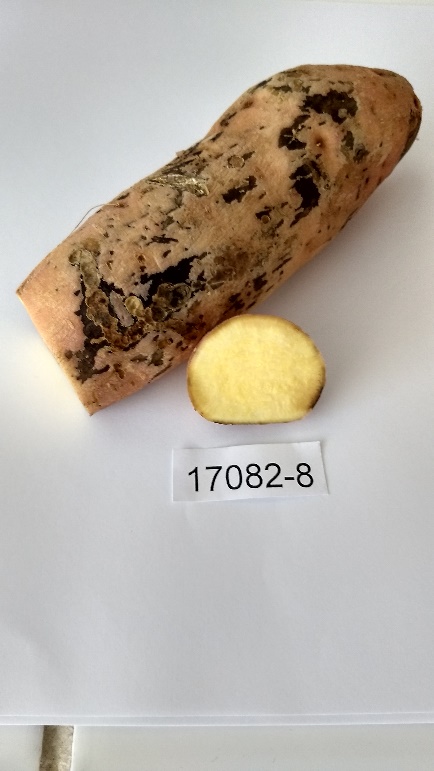 |
| 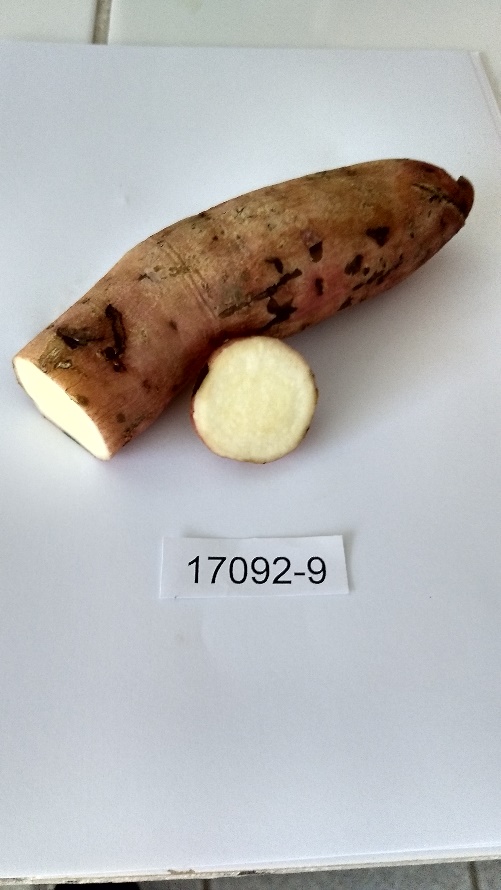 | 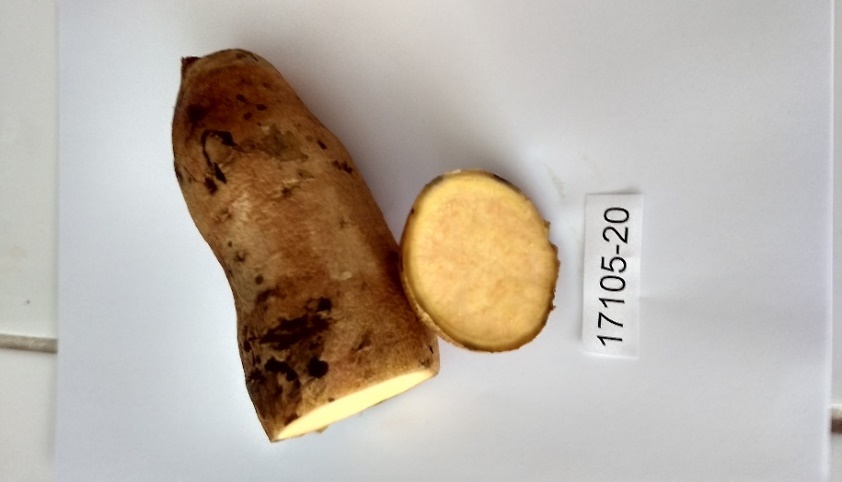 | 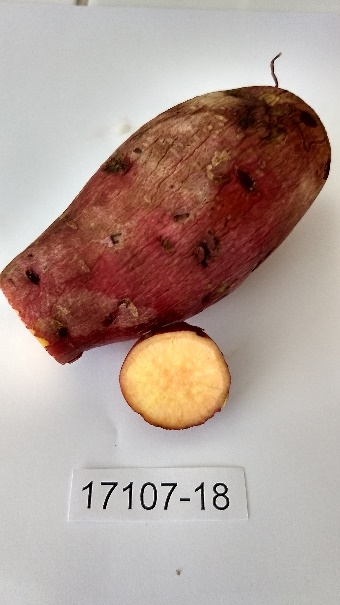 |
| 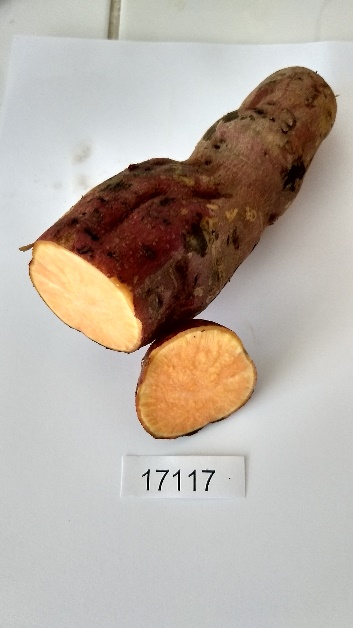 | 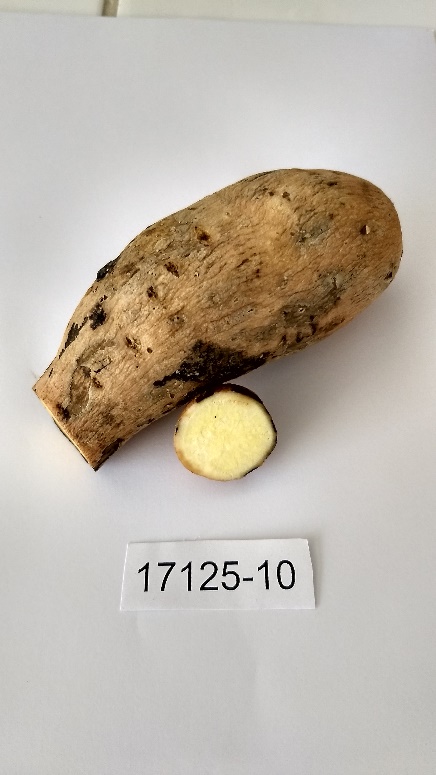 | 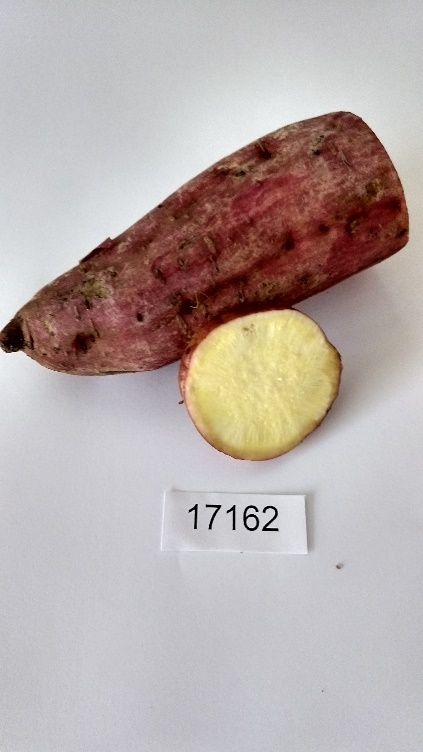 |

Supplementary Figure 1 - Registered and new varieties of sweet potato samples

**Supplementary Table 1** – First exploratory Factor Analysis: Sample correlations between the 16 input independent variables

|  | **Weight** | **Length** | **Width** | **Wa** | **M** | **Ev** | **P** | **L** | **Carb** | **Tdf** | **Ash** | **L*** | **a*** | **b*** | **C** |
| --- | --- | --- | --- | --- | --- | --- | --- | --- | --- | --- | --- | --- | --- | --- | --- |
| Length | 0.66 |  |  |  |  |  |  |  |  |  |  |  |  |  |  |
| Width | 0.56 | 0.18 |  |  |  |  |  |  |  |  |  |  |  |  |  |
| Wa | 0.14 | -0.07 | 0.45 |  |  |  |  |  |  |  |  |  |  |  |  |
| M | -0.01 | 0.08 | 0.16 | 0.01 |  |  |  |  |  |  |  |  |  |  |  |
| Ev | 0.09 | -0.07 | -0.05 | 0.12 | -0.94 |  |  |  |  |  |  |  |  |  |  |
| P | 0.02 | 0.09 | 0.02 | 0.07 | 0.34 | -0.29 |  |  |  |  |  |  |  |  |  |
| L | -0.07 | -0.16 | 0.15 | 0.30 | 0.16 | 0.07 | 0.22 |  |  |  |  |  |  |  |  |
| Carb | 0.06 | -0.05 | -0.09 | -0.04 | -0.83 | 0.78 | -0.77 | -0.33 |  |  |  |  |  |  |  |
| Tdf | 0.16 | -0.05 | 0.05 | 0.18 | -0.70 | 0.66 | -0.37 | -0.35 | 0.71 |  |  |  |  |  |  |
| Ash | 0.14 | -0.07 | 0.45 | 1.00 | 0.01 | 0.12 | 0.07 | 0.30 | -0.04 | 0.18 |  |  |  |  |  |
| L* | 0.19 | 0.32 | 0.01 | -0.38 | 0.13 | -0.03 | -0.06 | -0.11 | 0.04 | -0.10 | -0.38 |  |  |  |  |
| a* | -0.17 | -0.36 | -0.06 | 0.25 | -0.19 | 0.05 | 0.00 | 0.02 | 0.03 | 0.23 | 0.25 | -0.84 |  |  |  |
| b* | 0.02 | -0.10 | 0.10 | -0.08 | -0.06 | 0.02 | 0.02 | -0.05 | 0.01 | 0.18 | -0.08 | 0.41 | 0.05 |  |  |
| C | -0.12 | -0.36 | 0.10 | 0.20 | -0.18 | 0.06 | 0.06 | 0.01 | 0.01 | 0.29 | 0.20 | -0.33 | 0.67 | 0.71 |  |
| °h | -0.12 | -0.10 | -0.02 | 0.27 | -0.05 | 0.05 | 0.00 | 0.06 | 0.02 | -0.05 | 0.27 | -0.67 | 0.19 | -0.87 | -0.34 |

**Supplementary Table 2** – First exploratory Factor Analysis: Eigenvalues of 5 factors

| **Eigenvalues**  **Extraction: Principal components** | | | | |
| --- | --- | --- | --- | --- |
| **Factor** | **Eigenvalue** | **(%) Total**  **Variance** | **Cumulative**  **Eigenvalue** | **Cumulative (%)** |
| 1 | 3.83 | 23.93 | 3.83 | 23.93 |
| 2 | 3.27 | 20.42 | 7.10 | 44.35 |
| 3 | 2.46 | 15.35 | 9.55 | 59.70 |
| 4 | 2.41 | 15.04 | 11.96 | 74.74 |
| 5 | 1.27 | 7.91 | 13.22 | 82.64 |

**Supplementary Table 3** – First exploratory Factor Analysis: Relationship between response variables and factors

| **Factor Loadings (Varimax raw)**  **Extraction: Principal components** | | | | | |
| --- | --- | --- | --- | --- | --- |
| **Variables** | **Factor**  **1** | **Factor**  **2** | **Factor**  **3** | **Factor**  **4** | **Factor**  **5** |
| Weight | 0.06 | 0.15 | 0.05 | 0.20 | 0.87 |
| Length | -0.08 | 0.32 | -0.10 | -0.09 | 0.77 |
| Width | -0.08 | 0.02 | 0.14 | 0.58 | 0.54 |
| Wa | 0.05 | -0.24 | -0.09 | 0.90 | 0.10 |
| M | -0.91 | 0.11 | -0.02 | 0.02 | 0.07 |
| Ev | 0.89 | 0.09 | 0.00 | 0.19 | -0.11 |
| P | -0.62 | -0.06 | 0.06 | 0.15 | -0.01 |
| L | -0.23 | 0.24 | -0.01 | 0.63 | -0.42 |
| Carb | 0.97 | 0.02 | -0.03 | -0.12 | 0.05 |
| Tdf | 0.80 | -0.27 | 0.16 | 0.05 | 0.20 |
| Ash | 0.05 | -0.24 | -0.09 | 0.90 | 0.10 |
| L* | -0.01 | 0.83 | 0.44 | -0.23 | 0.15 |
| a* | 0.07 | -0.93 | 0.04 | 0.10 | -0.14 |
| b* | 0.04 | -0.03 | 0.99 | -0.02 | -0.02 |
| C* | 0.07 | -0.67 | 0.68 | 0.16 | -0.12 |
| °h | 0.02 | -0.28 | -0.90 | 0.17 | -0.07 |
| Expl. Var | 3.68 | 2.48 | 2.53 | 2.58 | 1.96 |
| Prp. Totl | 0.23 | 0.15 | 0.16 | 0.16 | 0.12 |

**Supplementary Figure 2 –** Eigenvalues of the 16 input independent variables in first exploratory Factor Analysis

**Supplementary Table 4** – First exploratory Factor Analysis: Communality between variables and factors

| **Communalities**  **Extraction: Principal components**  **Rotation: Varimax raw** | | | | | | |
| --- | --- | --- | --- | --- | --- | --- |
| **Variables** | **Factor 1** | **Factor 2** | **Factor 3** | **Factor 4** | **Factor 5** | **Multiple**  **R-Square** |
| Weight | 0.01 | 0.03 | 0.03 | 0.07 | 0.82 | 0.76 |
| Length | 0.01 | 0.11 | 0.12 | 0.13 | 0.73 | 0.66 |
| Width | 0.01 | 0.01 | 0.03 | 0.37 | 0.65 | 0.64 |
| Wa | 0.01 | 0.06 | 0.07 | 0.87 | 0.88 | 1.00 |
| M | 0.83 | 0.85 | 0.85 | 0.85 | 0.85 | 0.91 |
| Ev | 0.80 | 0.81 | 0.81 | 0.84 | 0.86 | 1.00 |
| P | 0.39 | 0.39 | 0.40 | 0.42 | 0.42 | 1.00 |
| L | 0.05 | 0.11 | 0.11 | 0.50 | 0.68 | 1.00 |
| Carb | 0.93 | 0.93 | 0.94 | 0.95 | 0.95 | 1.00 |
| Tdf | 0.64 | 0.72 | 0.74 | 0.74 | 0.79 | 0.65 |
| Ash | 0.01 | 0.06 | 0.07 | 0.87 | 0.88 | 1.00 |
| L* | 0.01 | 0.69 | 0.89 | 0.94 | 0.96 | 0.99 |
| a* | 0.01 | 0.87 | 0.87 | 0.88 | 0.90 | 0.99 |
| b* | 0.01 | 0.01 | 0.99 | 0.99 | 0.99 | 1.00 |
| C | 0.01 | 0.45 | 0.91 | 0.93 | 0.95 | 1.00 |
| °h | 0.01 | 0.08 | 0.89 | 0.92 | 0.92 | 0.99 |

S**upplementary Table 5** – Second exploratory Factor Analysis: Correlation between the 13 input independent variables

|  | **Weight** | | **Length** | | **Wa** | | **M** | **Ev** | **Carb** | **Tdf** | **Ash** | **L*** | **a*** | **b*** | **C** |
| --- | --- | --- | --- | --- | --- | --- | --- | --- | --- | --- | --- | --- | --- | --- | --- |
| Length | | 0.66 |  |  | |  | |  |  |  |  |  |  |  |  |
| Wa | | 0.14 | -0.07 |  | |  | |  |  |  |  |  |  |  |  |
| M | | -0.01 | 0.08 | 0.01 | |  | |  |  |  |  |  |  |  |  |
| Ev | | 0.09 | -0.07 | 0.12 | | -0.94 | |  |  |  |  |  |  |  |  |
| Carb | | 0.06 | -0.05 | -0.04 | | -0.83 | | 0.78 |  |  |  |  |  |  |  |
| Tdf | | 0.16 | -0.05 | 0.18 | | -0.70 | | 0.66 | 0.71 |  |  |  |  |  |  |
| Ash | | 0.14 | -0.07 | 1.00 | | 0.01 | | 0.12 | -0.04 | 0.18 |  |  |  |  |  |
| L* | | 0.19 | 0.32 | -0.38 | | 0.13 | | -0.03 | 0.04 | -0.10 | -0.38 |  |  |  |  |
| a* | | -0.17 | -0.36 | 0.25 | | -0.19 | | 0.05 | 0.03 | 0.23 | 0.25 | -0.84 |  |  |  |
| b* | | 0.02 | -0.10 | -0.08 | | -0.06 | | 0.02 | 0.01 | 0.18 | -0.08 | 0.41 | 0.05 |  |  |
| C | | -0.12 | -0.36 | 0.20 | | -0.18 | | 0.06 | 0.01 | 0.29 | 0.20 | -0.33 | 0.67 | 0.71 |  |
| °h | | -0.12 | -0.10 | 0.27 | | -0.05 | | 0.05 | 0.02 | -0.05 | 0.27 | -0.67 | 0.19 | -0.87 | -0.34 |

**Supplementary Table 6** – Second exploratory Factor Analysis: Eigenvalues

| **Eigenvalues**  **Extraction: Principal components** | | | | |
| --- | --- | --- | --- | --- |
| **Factor** | **Eigenvalue** | **% Total**  **Variance** | **Cumulative**  **Eigenvalue** | **Cumulative (%)** |
| 1 | 3.64 | 27.96 | 3.64 | 27.96 |
| 2 | 2.99 | 23.04 | 6.63 | 51.00 |
| 3 | 2.42 | 18.61 | 9.05 | 69.61 |
| 4 | 1.87 | 14.42 | 10.92 | 84.02 |
| 5 | 1.05 | 8.06 | 11.97 | 92.08 |

**Supplementary Table 7** – Second exploratory Factor Analysis: Relationship between variables and calculated factors

| **Factor Loadings (Varimax raw) Extraction: Principal components** | | | | | |
| --- | --- | --- | --- | --- | --- |
| **Variables** | **Factor 1** | **Factor 2** | **Factor 3** | **Factor 4** | **Factor 5** |
| Weight | 0.07 | 0.07 | -0.06 | 0.14 | 0.91 |
| Length | -0.07 | 0.25 | 0.08 | -0.08 | 0.88 |
| Wa | 0.02 | -0.14 | 0.06 | 0.99 | 0.03 |
| M | -0.95 | 0.13 | 0.01 | 0.05 | 0.03 |
| Ev | 0.94 | 0.04 | 0.03 | 0.10 | -0.02 |
| Carb | 0.92 | 0.06 | 0.03 | -0.06 | 0.00 |
| Tdf | 0.81 | -0.18 | -0.18 | 0.15 | 0.11 |
| Ash | 0.02 | -0.14 | 0.06 | 0.99 | 0.03 |
| L* | -0.02 | 0.86 | -0.43 | -0.23 | 0.15 |
| a* | 0.07 | -0.96 | -0.06 | 0.11 | -0.12 |
| b* | 0.04 | -0.01 | -0.99 | -0.03 | -0.04 |
| C | 0.08 | -0.65 | -0.68 | 0.16 | -0.16 |
| °h | 0.03 | -0.27 | 0.90 | 0.19 | -0.10 |
| Expl. Var | 3.32 | 2.32 | 2.50 | 2.14 | 1.69 |
| Prp. Totl | 0.26 | 0.18 | 0.19 | 0.16 | 0.13 |

**Supplementary Figure 3** – Second Exploratory Factor Analysis: Eigenvalues of the 13 input independent variables

**Supplementary Table 8** – Second exploratory Factor Analysis: Factors communality

| **Communalities**  **Extraction: Principal components**  **Rotation: Varimax raw** | | | | | | |
| --- | --- | --- | --- | --- | --- | --- |
| **Variables** | **Factor 1** | **Factor 2** | **Factor 3** | **Factor 4** | **Factor 5** | **Multiple**  **R-Square** |
| Weight | 0.01 | 0.01 | 0.01 | 0.03 | 0.87 | 0.57 |
| Length | 0.00 | 0.07 | 0.07 | 0.08 | 0.85 | 0.63 |
| Wa | 0.00 | 0.02 | 0.02 | 1.00 | 1.00 | 1.00 |
| M | 0.91 | 0.93 | 0.93 | 0.93 | 0.93 | 0.95 |
| Ev | 0.88 | 0.88 | 0.88 | 0.89 | 0.89 | 0.94 |
| Carb | 0.85 | 0.86 | 0.86 | 0.86 | 0.86 | 0.80 |
| Tdf | 0.66 | 0.69 | 0.72 | 0.75 | 0.76 | 0.65 |
| Ash | 0.00 | 0.02 | 0.02 | 1.00 | 1.00 | 1.00 |
| L* | 0.00 | 0.74 | 0.92 | 0.97 | 0.99 | 0.99 |
| a* | 0.01 | 0.92 | 0.93 | 0.94 | 0.95 | 0.98 |
| b* | 0.00 | 0.00 | 0.98 | 0.99 | 0.99 | 0.94 |
| C | 0.01 | 0.43 | 0.90 | 0.93 | 0.95 | 0.94 |
| °h | 0.00 | 0.07 | 0.88 | 0.92 | 0.93 | 0.96 |

**Supplementary Table 9** – Principal Component Analysis: Mean and standard deviation of standardized variables

| **Summary statistics** | | |
| --- | --- | --- |
| **Variables** | **Mean** | **Standard deviation** |
| Weight | 0.13 | 0.79 |
| Length | 0.07 | 0.90 |
| Wa | -0.02 | 1.09 |
| M | 0.03 | 0.93 |
| Ev | 0.02 | 0.92 |
| Carb | -0.03 | 0.98 |
| Tdf | -0.01 | 1.00 |
| Ash | -0.02 | 1.09 |
| L* | -0.01 | 1.00 |
| a* | 0.01 | 1.01 |
| b* | 0.01 | 1.02 |
| C | 0.01 | 1.01 |
| °h | -0.01 | 1.00 |

**Supplementary Table 10** – Principal Component Analysis: Eigenvalues of the main components

| **Eigenvalues of correlation matrix and related statistics**  **Active variables only** | | | | |
| --- | --- | --- | --- | --- |
| **Principal**  **Components** | **Eigenvalue** | **% Total**  **variance** | **Cumulative**  **Eigenvalue** | **Cumulative (%)** |
| 1 | 3.64 | 27.96 | 3.64 | 27.96 |
| 2 | 2.99 | 23.04 | 6.63 | 51.00 |
| 3 | 2.42 | 18.61 | 9.05 | 69.61 |
| 4 | 1.87 | 14.42 | 10.92 | 84.02 |
| 5 | 1.05 | 8.06 | 11.97 | 92.08 |
| 6 | 0.36 | 2.77 | 12.33 | 94.85 |
| 7 | 0.28 | 2.18 | 12.61 | 97.03 |
| 8 | 0.19 | 1.50 | 12.81 | 98.53 |
| 9 | 0.16 | 1.21 | 12.97 | 99.74 |
| 10 | 0.03 | 0.21 | 12.99 | 99.95 |
| 11 | 0.01 | 0.04 | 13.00 | 100.00 |
| 12 | 0.00 | 0.00 | 13.00 | 100.00 |

**Supplementary Table 11** – Principal Component Analysis: Coordinates of each factor as a function of the variables

| **Factor coordinates of the variables based on correlations** | | | | |
| --- | --- | --- | --- | --- |
| **Variables** | **PC 1** | **PC 2** | **PC 3** | **PC 4** |
| Weight | -0.05 | -0.25 | -0.30 | -0.71 |
| Lengtht | -0.33 | -0.29 | -0.42 | -0.53 |
| Wa | 0.43 | 0.51 | -0.04 | -0.65 |
| M | -0.80 | 0.47 | 0.21 | -0.18 |
| Ev | 0.75 | -0.46 | -0.30 | 0.04 |
| Carb | 0.67 | -0.54 | -0.30 | 0.15 |
| Tdf | 0.77 | -0.39 | -0.06 | -0.11 |
| Ash | 0.43 | 0.51 | -0.04 | -0.65 |
| L* | -0.53 | -0.75 | 0.01 | -0.19 |
| a* | 0.59 | 0.48 | 0.40 | 0.13 |
| b* | 0.05 | -0.49 | 0.83 | -0.25 |
| C | 0.48 | 0.06 | 0.82 | -0.11 |
| °h | 0.22 | 0.65 | -0.63 | 0.24 |

**Supplementary Table 12** – Principal Component Analysis: Sample scores

| **Factor coordinates of cases based on correlations**  **Labelling variable: SAMPLES** | | | | | |
| --- | --- | --- | --- | --- | --- |
| **Number** | **Samples** | **PC 1** | **PC 2** | **PC 3** | **PC 4** |
| 1 | SCS367 Favorita | -0.50 | 1.84 | 2.72 | -0.01 |
| 1 | SCS367 Favorita | -0.43 | 0.55 | 2.81 | 2.86 |
| 2 | SCS368 Ituporanga | 0.15 | -0.48 | -0.32 | 0.98 |
| 2 | SCS368 Ituporanga | -0.40 | 0.10 | -0.74 | -1.28 |
| 3 | SCS369 Águas Negras | -1.99 | 0.43 | -0.99 | -1.02 |
| 3 | SCS369 Águas Negras | -2.25 | 0.32 | -1.59 | -1.82 |
| 4 | SCS370 Luiza | 3.11 | 4.51 | -3.14 | 1.72 |
| 4 | SCS370 Luiza | 2.42 | 5.52 | -2.85 | 1.19 |
| 5 | SCS371 Katiy | 0.09 | 0.24 | -0.85 | 0.04 |
| 5 | SCS371 Katiy | -0.48 | -0.81 | -2.12 | -0.64 |
| 6 | SCS372 Marina | 3.88 | -2.40 | -0.28 | 0.57 |
| 6 | SCS372 Marina | 2.90 | -2.34 | -1.00 | -1.33 |
| 7 | Darci | -1.74 | -0.41 | -1.47 | 0.19 |
| 7 | Darci | -2.95 | -1.10 | -1.27 | 1.77 |
| 8 | Leandro | -2.46 | 0.64 | -0.52 | -0.86 |
| 8 | Leandro | -1.80 | -0.75 | -0.38 | 2.48 |
| 9 | 17007-15 | 2.55 | 1.25 | 2.12 | -2.23 |
| 9 | 17007-15 | 1.22 | 1.81 | 2.53 | -0.94 |
| 10 | 17025-13 | 3.15 | -2.98 | 0.53 | 1.68 |
| 10 | 17025-13 | 2.88 | -0.73 | 1.28 | 1.13 |
| 11 | 17052 | -0.71 | 0.90 | 1.06 | -0.97 |
| 11 | 17052 | -1.84 | 1.01 | 1.27 | -0.10 |
| 12 | 17082-8 | 0.36 | -1.12 | 0.54 | 0.06 |
| 12 | 17082-8 | 0.37 | -1.86 | -0.06 | -0.07 |
| 13 | 17092-9 | 0.75 | -1.34 | -1.92 | -0.03 |
| 13 | 17092-9 | -0.49 | -1.26 | -2.07 | -0.49 |
| 14 | 17105-20 | -0.19 | -0.52 | 0.83 | 0.75 |
| 14 | 17105-20 | -0.88 | 0.08 | 1.81 | 2.48 |
| 15 | 17107-18 | 1.70 | -2.19 | -0.17 | -1.14 |
| 15 | 17107-18 | 1.41 | -0.31 | 1.04 | 0.01 |
| 16 | 17117 | -1.26 | 1.64 | 2.14 | -1.01 |
| 16 | 17117 | -0.23 | 1.54 | 1.61 | -1.34 |
| 17 | 17125-10 | -0.18 | -0.35 | 0.19 | -1.62 |
| 17 | 17125-10 | -0.08 | -0.13 | -0.18 | -2.50 |
| 18 | 17162 | -3.47 | -0.65 | 0.20 | 1.64 |
| 18 | 17162 | -2.62 | -0.67 | -0.75 | -0.17 |

**Supplementary Figure 4** – Principal Component Analysis: Cumulative percentage of eigenvalues in relation to the 13 input independent variables

**Supplementary Table 13** – Principal Component Analysis: Communality correlations between variables and factors

| **Communalities. based on correlations** | | | | |
| --- | --- | --- | --- | --- |
| **Variables** | **PC1** | **PC2** | **PC3** | **PC4** |
| Weight | 0.00 | 0.06 | 0.15 | 0.66 |
| Length | 0.11 | 0.19 | 0.37 | 0.66 |
| Wa | 0.19 | 0.45 | 0.45 | 0.87 |
| M | 0.63 | 0.85 | 0.90 | 0.93 |
| Ev | 0.57 | 0.78 | 0.87 | 0.87 |
| Carb | 0.45 | 0.74 | 0.84 | 0.86 |
| Tdf | 0.59 | 0.74 | 0.74 | 0.76 |
| Ash | 0.19 | 0.45 | 0.45 | 0.87 |
| L* | 0.29 | 0.84 | 0.84 | 0.88 |
| a* | 0.35 | 0.58 | 0.74 | 0.75 |
| b* | 0.00 | 0.24 | 0.92 | 0.98 |
| C | 0.23 | 0.23 | 0.91 | 0.92 |
